# Messages Do Diffuse Faster than Messengers: Reconciling Disparate Estimates of the Morphogen Bicoid Diffusion Coefficient

**DOI:** 10.1371/journal.pcbi.1003629

**Published:** 2014-06-05

**Authors:** Lorena Sigaut, John E. Pearson, Alejandro Colman-Lerner, Silvina Ponce Dawson

**Affiliations:** 1 Departamento de Física and IFIBA, FCEN-UBA - CONICET, Buenos Aires, Argentina; 2 Theoretical Biology and Biophysics, Los Alamos National Laboratory, Los Alamos, New Mexico, United States of America; 3 Departamento de Fisiología, Biología Molecular y Celular and IFIBYNE, CONICET, FCEN-UBA, Buenos Aires, Argentina; McMaster University, Canada

## Abstract

The gradient of Bicoid (Bcd) is key for the establishment of the anterior-posterior axis in Drosophila embryos. The gradient properties are compatible with the *SDD* model in which Bcd is *synthesized* at the anterior pole and then *diffuses* into the embryo and is *degraded* with a characteristic time. Within this model, the Bcd diffusion coefficient is critical to set the timescale of gradient formation. This coefficient has been measured using two optical techniques, *Fluorescence Recovery After Photobleaching* (**FRAP**) and *Fluorescence Correlation Spectroscopy* (**FCS**), obtaining estimates in which the **FCS** value is an order of magnitude larger than the **FRAP** one. This discrepancy raises the following questions: which estimate is "correct''; what is the reason for the disparity; and can the SDD model explain Bcd gradient formation within the experimentally observed times? In this paper, we use a simple biophysical model in which Bcd diffuses and interacts with binding sites to show that both the **FRAP** and the **FCS** estimates may be correct and compatible with the observed timescale of gradient formation. The discrepancy arises from the fact that **FCS** and **FRAP** report on different *effective* (concentration dependent) diffusion coefficients, one of which describes the spreading rate of the individual Bcd molecules (the *messengers*) and the other one that of their concentration (the *message*). The latter is the one that is more relevant for the gradient establishment and is compatible with its formation within the experimentally observed times.

## Author Summary

Understanding the mechanisms by which equivalent cells develop into different body parts is a fundamental question in biology. One well-studied example is the patterning along the anterior-posterior axis of *Drosophila melanogaster* embryos for which the spatial gradient of the protein Bicoid is determinant. The localized production of Bicoid is implicated in its inhomogeneous distribution. Diffusion then determines the time and spatial scales of the gradient as it is formed. Estimates of Bicoid diffusion coefficients made with the optical techniques, **FRAP** and **FCS** resulted in largely different values, one of which was too slow to account for the observed time of gradient formation. In this paper, we present a model in which Bicoid diffuses and interacts with binding sites so that its transport is described by a "single molecule'' and a "collective'' diffusion coefficient. The latter can be arbitrarily larger than the former coefficient and sets the rate for bulk processes such as the formation of the gradient. In this way we obtain a self-consistent picture in which the **FRAP** and **FCS** estimates are accurate and where the gradient can be established within the experimentally observed times.

## Introduction

**Figure 1:**
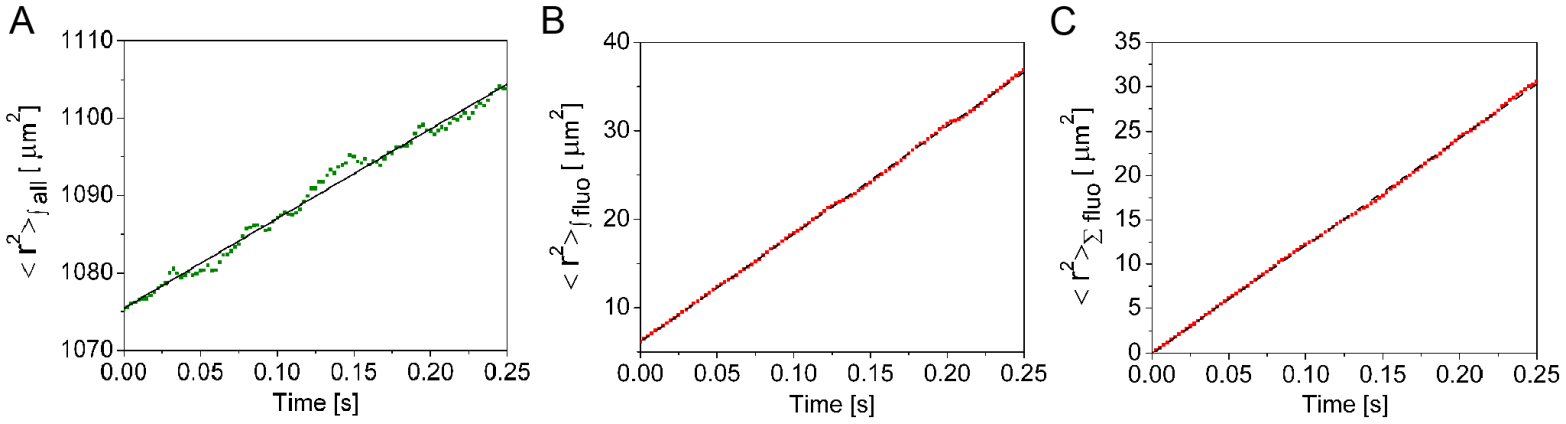
Second moments of particle distributions and mean square displacements obtained from a simulation of freely diffusing particles. The data for this figure (shown in green and red) comes from the simulation of Video 0.1 which corresponds to a system of particles that diffuse with 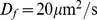 in the absence of binding sites. The simulation starts with an equilibrium situation that is perturbed by adding 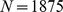 fluorescent particles to the the central 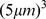 cube of the 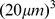 simulation volume. In this figure we characterize the rate at which the deviations with respect to equilibrium of the concentrations of all particles and of the added ones spread out with time by means of second moments. We compare these second moments with the **MSD** of the added particles. Please notice that we are not plotting the mean square displacements in **A**) and **B**) but a quantity (the second moment) that depends linearly with the time lag with the same slope as the mean square displacement. For more details see Materials and Methods. **A**: 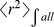 (shown in green) computed using Eq. (12) with 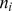 the number of all particles in the 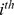 box. Linear fit (shown in black). **B**: 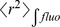 (shown in red) computed using Eq. (12) with 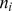 the number of fluorescent particles in the 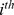 box. Linear fit (shown in black). **C**: The mean of the squared displacements of the added particles (shown in red) computed using Eq. (11). Linear fit (shown in black). As explained in supplementary text S3 the diffusion coefficient, 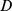, can be estimated by taking 1/6 of the slope of the fitting curves. In this case the three estimates yield 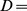 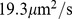 (A), 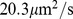 (B) and 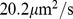 (C). The second moment shown in **A**) corresponds to the "collective diffusion coefficient'', the one in **C**) to the "single molecule diffusion coefficient'' and the one in **B**) could be called 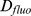. According to the theory all three should coincide in the case of freely diffusing particles and this is reflected in this figure.

**Figure 2:**
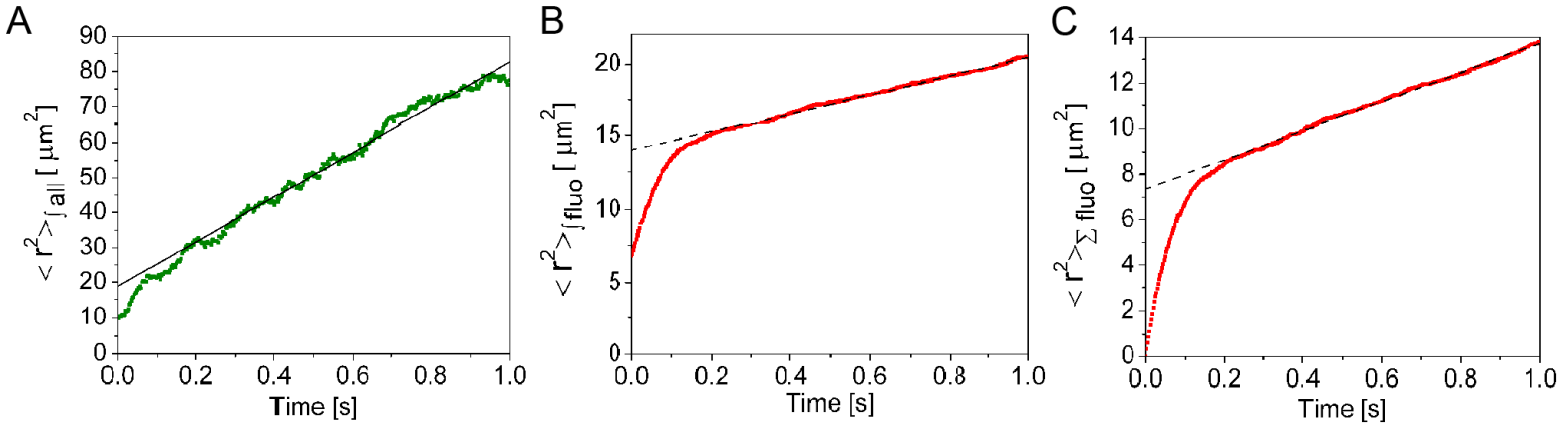
Second moments of particle distributions and mean square displacements obtained from a simulation of particles that diffuse and react with immobile binding sites. Similar to Fig. 1 but for the simulation of Video S2 which corresponds to a system of particles that diffuse with 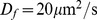 and react with immobile binding sites according to Eq. (1). The simulation parameters are such that the effective diffusion coefficients defined in Eq. (2) are 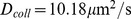 and 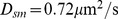. As in the simulation with the freely diffusing particles, the simulation starts when a bolus of fluorescent particles is added to a background of (non-fluorescent) particles that are initially spatially uniform and in chemical equilibrium with the binding sites. Also in this case we compute two second moments and the averaged mean square displacement of the added particles to quantify relevant properties of the simulation. For more details see Materials and Methods. **A**: 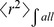 (shown in gren) computed using Eq. (12) with 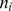 the number of all particles in the 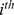 box. Linear fit (shown in black). **B**: 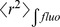 (shown in red) computed using Eq. (12) with 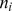 the number of fluorescent particles in the 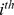 box. Linear fit (shown in black). **C**: The mean of the squared displacements of the added particles (shown in red) computed using Eq. (11). Linear fit (shown in black). As explained in supplementary text 0.3, in this case, the diffusive behavior sets in after a transient. Once this behavior is reached, diffusion coefficients, 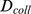, 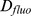 and 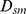, can also be estimated by taking 1/6 of the slope of the three fitting curves. Differently from the situation of freely diffusing particles, in this case, the estimates differ from one another. They yield A: 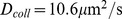, B: 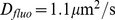, C: 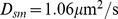. According to the theory (see supplementary text 0.3), 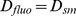 and 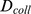 and 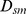 should be given by Eqs. (2). In fact, the values derived from the simulation satisfy 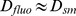 and are pretty close to the theoretical values, 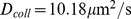 and 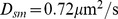.

Diffusion is a key factor underlying many physiological processes among them, the formation of morphogen gradients. Having reliable estimates of diffusion rates in cells is thus of great relevance. Optical techniques provide a means to obtain such estimates. A difficulty with their direct application in cells and embryos is that free diffusion, as first considered by Einstein[1], [2], rarely occurs in living organisms [3], [4]. In particular, in many occasions binding/unbinding processes hinder transport. When the resulting net transport is observed over a long enough time it usually recovers the properties of (normal) diffusion but with a diffusion coefficient that depends on concentrations and on the rates of binding/unbinding as well. A single species, 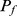, that reacts with slowly diffusing or immobile binding sites, 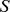, to form a complex 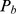, 
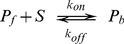
(1)has two distinct diffusion coefficients: a "collective'' one, 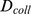, that governs the rate at which concentrations spread, and a "single molecule'' one, 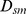, that governs the rate at which the mean squared displacement of the individual particles increase with time [5]. Both types of coefficients are weighted averages of the free diffusion coefficients, 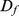, of the molecules, 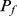, and that of the binding sites, 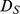, that depend on the concentrations of the species involved. In the case of the scheme given by Eqs. (1) or (5) they read:
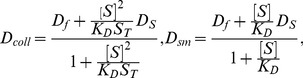
(2)where 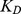 is the dissociation constant of the binding/unbinding process and 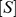 and 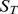 are the unbound and total binding sites concentrations, respectively. If the molecules do not react but only diffuse freely within a simple solvent, it is 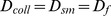. If they also bind/unbind to/from slowly moving sites, the ratio 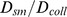 can be arbitrarily small [5]. We illustrate the difference between a situation with freely diffusing particles and with particles that diffuse and react by means of Videos S1 and S2, respectively. In both these simulations a bolus of fluorescent particles is initially added to an equilibrium situation. The subsequent spread of the deviation of the concentration of all the particles with respect to equilibrium (left most panels), of the concentration of added particles (center panels) and of the added particles (right most panels) are shown in these Videos. The rate at which these three quantities spread out with time are characterized, respectively, by the second moment of the distribution of all particles, the second moment of the distribution of added particles and by the mean square displacements of the added particles (see Materials and Methods). These are shown in Figs. 1 and 2. In both simulations, the quantities shown in the figures eventually scale linearly with time and diffusion coefficients can be estimated from the slopes (see Materials and Methods and supplementary text S3). We observe that for the freely diffusing particles all three slopes yield the same diffusion coefficient to within a few percent which coincides with the free coefficient of the particles (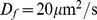 in the simulation). In the case in which the particles interact with the binding sites the coefficient derived from the slope of the second moment of the distribution of all particles (Fig. 2 A) is an order of magnitude larger than the other two which coincide between themselves. The former corresponds to 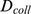 (Eq. (2) gives 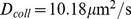 for the simulation parameters) and the other two to 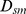 (Eq. (2) gives 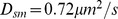 for the simulation parameters). Video S2 and Fig. 2 show that the spreading of the individual particles and that of the deviation with respect to equilibrium of the total particle concentration are eventually diffusive but with two different (effective) diffusion coefficients in the presence of binding/unbinding (for more details see supplementary text S1). The existence of one coefficient ruling the diffusion of individual particles and another one ruling the decay of concentration gradients also occurs in the context of non-ideal solutions [6] particularly those involving polymers [3], [7]. The combination of free diffusion and binding/unbinding processes can also result in what is called *anomalous diffusion* [3], [4], [8], [9]. The defining property of this type of transport is that, differently from normal diffusion, the mean square displacement of a molecule is not proportional to the time elapsed. In this Introduction we will limit the description to situations in which the observed transport has the properties of normal diffusion. We discuss the properties of free, anomalous and effective diffusion in more detail in supplementary text S1.

**Figure 3:**
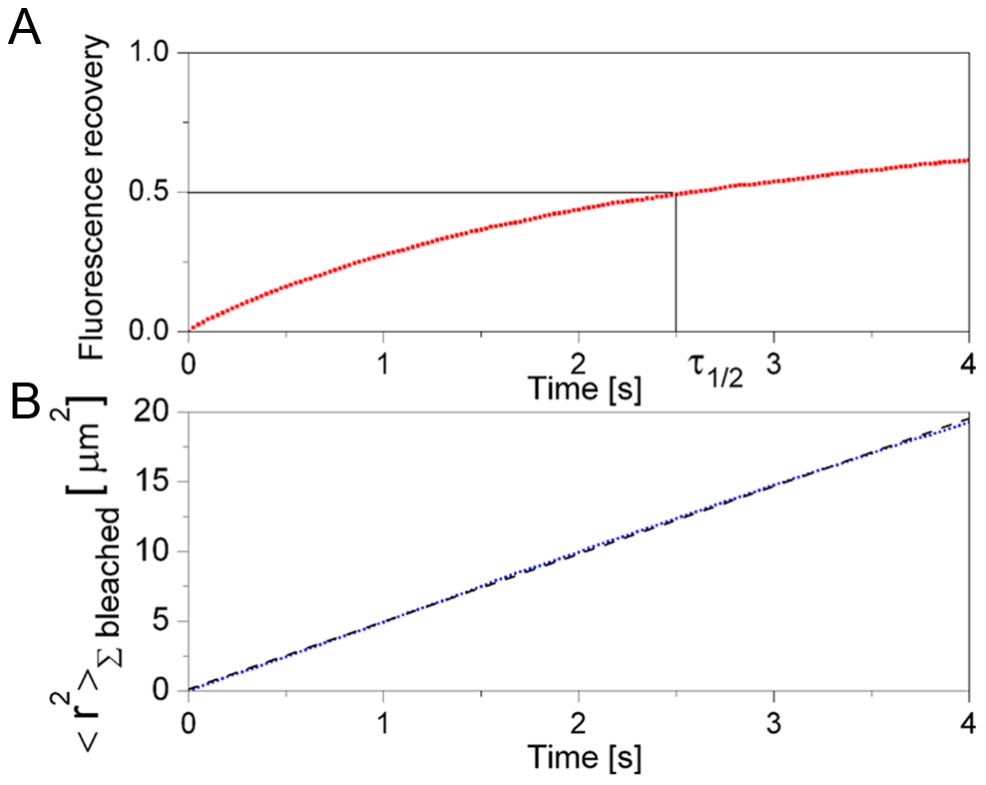
Simulated FRAP-like experiment. **A**: Recovery of relative fluorescence, 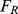, given by Eq. (14) (shown in red), obtained from the simulated **FRAP** experiment depicted in Video 0.3. For the initial conditions of the simulation (a totally bleached spherical volume of radius 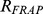) the half recovery time, 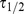, (*i.e.* the time at which 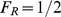) is related to the diffusion coefficient by 
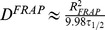
. From the simulation we obtain 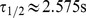. Using 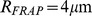 we derive 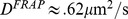. **B**: Single molecule **MSD**, 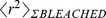, computed using Eq. (11) for the bleached particles, as a function of time (shown in blue) and linear fit (shown in black). The slope of the fitting curve is 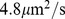 which yields an estimated diffusion coefficient of 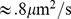.

In the effective diffusion regime, the optical techniques, fluorescence recovery after photobleaching (**FRAP**), and fluorescence correlation spectroscopy (**FCS**), provide information on the effective diffusion coefficients. **FRAP** is an optical technique that is commonly used to estimate the diffusion rate of fluorescently labeled proteins in cells [10], [11]. In **FRAP** the fluorescence is photobleached inside a small region. By measuring the time it takes for the fluorescence to recover the transport rate of the fluorescent species can be estimated. When the fluorescent species diffuses and undergoes binding/unbinding reactions and the transport is effectively diffusive **FRAP** prescribes the single molecule coefficient [5], [6], [12]–[14]. We illustrate this in Video S3 where we show the simulation of a **FRAP** -like experiment. The interaction between the binding sites and the particles is the same in this simulation as in Video S2. The circle that is predominant at the beginning is the (projection into 2 space dimensions of) the **FRAP** volume. The particles in the **FRAP** volume that are bleached are shown as blue at all times. The unbleached particles inside the **FRAP** volume are shown as red. The unbleached particles outside the **FRAP** volume are not shown. Fig. 3 A shows the recovery curve and the time of 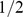 recovery for this "experiment''. From the recovery curve we estimate 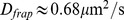. The **MSD** graph yields 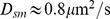. We see that **FRAP** and particle tracking give diffusion coefficients in reasonable agreement with that of 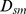 in Eq. (2) (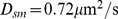). These estimated effective diffusion coefficients are in rough accord with the single molecule coefficients obtained in the particle bolus simulation (Figs. 2B, C) and are an order of magnitude smaller than the collective diffusion derived from the same simulation (Fig. 2A). **FCS** is also commonly used to estimate diffusion coefficients of fluorescently labeled proteins. When the fluorescent proteins diffuse and react with other species **FCS** can give information on both effective coefficients [12]. Both **FRAP** and **FCS** produce time-dependent data whose interpretation requires an underlying mechanistic model. By fitting the experimental data to functions derived from the model, one may obtain estimates of model parameters. The choice of the mechanistic model is especially important when diffusion and binding/unbinding processes are involved since the fitted parameters need not correspond to fixed model parameters (*e.g.*, free diffusion coefficients), but instead may be functions of space or time dependent quantities (*e.g.* concentrations). **FCS** and **FRAP** have been used to estimate the diffusion coefficient of the morphogen Bicoid in *Drosophila melanogaster* embryos giving values such that the one obtained with **FCS** is an order of magnitude larger than the one obtained with **FRAP** [15], [16]. In this paper we analyze these results using an underlying mechanistic model that provides a clear distinction between fixed parameters and model variables (see Materials and Methods). In this way we determine a consistent set of model parameters that explains the difference in the Bicoid diffusion coefficients obtained with **FRAP** and **FCS**.

Bicoid (Bcd) is a key morphogen for the organization of the anterior-posterior axis in *Drosophila* embryos [17], [18]. The inhomogeneous distribution of its concentration induces the differential expression of certain genes determining the embryo body plan along the axis [18]. This patterning starts with the deposition of maternal cues, among them the transcription factor Bcd, into the developing egg. About 2 hours after egg deposition Bcd is unevenly distributed in the embryo with a gradient of concentration that decays exponentially from the anterior to the posterior end. This gradient becomes stable within 80 min (at 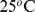) after deposition and remains stable during the next several nuclei divisions [16]. The question naturally arises as to what are the mechanisms by which this gradient is established so rapidly and precisely. To answer this question it is necessary to determine how the Bcd distribution depends on the localization and dynamics of the underlying Bcd mRNA and how Bcd is transported and affected by other processes inside the cell. The exponential distribution of the Bcd concentration is consistent with the so-called *SDD* model in which the protein is *synthesized* at the anterior end and subsequently *diffuses* and is *degraded* throughout the embryo [17], [18]. Within this model the Bcd diffusion coefficient is key to set the timescale over which the Bcd gradient forms and becomes stable. Gregor et al. [16] estimated the Bcd diffusion coefficient using **FRAP** during mitosis in embryos that expressed Bcd-EGFP (Bicoid fused to eGFP). Surprisingly, their estimate 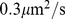) was an order of magnitude lower than the value that is necessary in the SDD model to account for the formation of a stable gradient within the observed times. Consequently, Spirov et al. [19] suggested an alternative model for the Bcd gradient formation and stabilization. Namely, they argued that the Bcd gradient is the reflection of an underlying bcd mRNA gradient. Later on, the diffusion coefficient of Bcd was again estimated but using **FCS** in the cytoplasm [15] and inside nuclei [20] of Bcd-EGFP expressing embryos. These experiments yield a set of values one of which was as fast as needed by the SDD model to explain the establishment of a stable gradient within the experimentally observed time. In view of this new estimate, the SDD cannot be discarded without first reconciling the two contrasting measurements of Bcd diffusion. Further support for the SDD model came from the results of Little et al. [21] according to which 90% of the Bcd mRNA is located within the anterior 20% of the embryo at any given time. Furthermore, including the observed mRNA gradient in an extended version of the SDD model, these authors concluded that the mRNA gradient could not account by itself for the protein gradient dynamics so that Bcd movement was necessary for the formation of its gradient. In view of these results, having reliable estimates of the rate at which Bcd diffuses in embryos becomes again most relevant. Abu-Arish et al. [15] not only estimated this coefficient using **FCS** but also performed **FRAP** experiments which yield a value of the same order of magnitude as the one obtained by Gregor et al. [16]. The question then arises as to what is the rate at which Bcd diffuses, the one given by **FRAP** or the one given by **FCS**? In order to answer this question it is necessary to understand why these values are so different. Abu-Arish et al. argued that their **FRAP** estimate was only a lower bound of the actual Bcd diffusion coefficient since the **FRAP** recovery half-time, 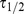, they determined was of the order of the photobleaching time, 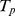. However, as discussed in supplementary text S2, we do not expect the estimate determined by **FRAP** to be so far off from the actual value only because 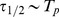. Our explanation of the discrepancy between the **FCS** and **FRAP** estimates is based on our demonstration that these two techniques report different effective coefficients (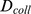, or 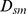) when probing the transport of a substance that does not diffuse freely but also undergoes binding and unbinding [12]. Since the collective, 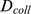, and the single molecule, 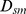, coefficients can be very different for molecules that diffuse and interact with slowly moving partners [5], we explain the disparate Bcd diffusion estimates by hypothesizing the existence of a significant pool of Bcd interacting molecules at the cortex during embryo development. Given that Bcd has demonstrated physical interactions with several proteins [22]–[24], and that it is able to bind specific mRNA species in the cytoplasm [25] it is reasonable to assume that Bcd does undergo binding/unbinding processes as it diffuses within the embryo. One argument in favor of this assumption is that **FCS** experiments performed using NLS-EGFP (a construct with a short nuclear localization signal and a GFP tag identical to that in Bcd-EGFP but that should diffuse freely in the cytoplasm [15]) yielded a larger diffusion coefficient than the one obtained for Bcd-EGFP with a difference that cannot be accounted for by the smaller size of NLS-EGFP relative to Bcd-EGFP.

In the present work we combine various published experimental results and interpret them within a biophysical model in which Bcd molecules interact with a single type of binding sites and can be fluorescent or not depending on EGFP maturation. Building upon the results of Pando et al. [5] and Sigaut et al. [12] we obtain a consistent set of values for the free diffusion coefficients, concentrations and dissociation constant of the model species that explains the difference in the Bcd-EGFP (effective) diffusion coefficients determined with **FRAP** and **FCS**. In view of the different physical meanings of the coefficients reported by **FRAP** and **FCS** we also conclude that the experimentally observed time it takes for the Bcd gradient formation is compatible with the SDD model.

## Results

We use the simple biophysical model described in Materials and Methods to interpret the results of the **FCS** experiments of Abu-Arish et al. [15] performed to probe the transport of Bcd-EGFP in the cortical cytoplasm of the anterior region of embryos during interphases of cycles 12–14 and those of Porcher et al. [20] performed in anterior nuclei during cycles 13 and 14. In our model, fluorescent and non-fluorescent Bcd-EGFP molecules coexist, diffuse with free coefficient, 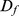, and interact with dissociation constant, 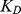, with a single type of binding sites, 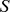, that diffuse with free coefficient 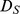 (see Table 1 for a complete list of symbols of the model). For the analysis we map the correlation times derived from fits to the auto-correlation function (ACF) of the fluorescence fluctuations presented in Refs. [15], [20] to analytic expressions that we derive for our model in terms of model parameters. Abu-Arish et al. [15] tried fits with different numbers of correlation times (or components). Those that provided the best results had two or three. In Ref. [20] only the results of fits with two components were presented. Our analytic ACF also has two or three components depending on whether the traps are immobile (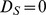) or not with times that correspond to the effective coefficients, 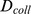 and 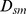, defined in Eq. (2) and to the free coefficient of the traps, 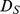 (in case it is not zero). The mapping between the parameters of our ACF and those of the fits of Refs. [15], [20] is done by associating the components depending on the relative ordering of the times which in our case is 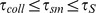 (see Materials and Methods). We also analyze the results of **FCS** experiments performed using NLS-EGFP [15], [20]. Assuming that this construct does not interact with binding sites, the free Bcd-EGFP diffusion coefficient, 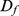, can be derived from the fits to these experiments taking into account a conversion factor due to the different molecular weights of both molecules. Thus, from the analysis of the correlation times derived from **FCS** experiments we determine both free and effective diffusion coefficients and, using Eqs. (2) and other properties of the model, concentrations and the dissociation constant of the reaction between Bcd and its putative binding sites. Our approach allows us to separate fixed parameters and variables so that we can analyze experiments performed under other conditions for which the variables can take on other values. In particular, we analyze the fluorescence recovery time obtained in the **FRAP** experiments of Abu-Arish et al. [15] and of Gregor et al. [16] which were performed during the mitosis following nuclear cycles 12 or 13 and determine that they are consistent with the parameters derived from the **FCS** experiments. We show the results obtained and some consistency tests in what follows. For more details we refer the reader to supplementary text S2.

**Table 1:** List of main symbols used in this paper.

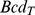	Total Bcd concentration
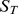	Total concentration of binding sites
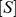	Concentration of unbound binding sites
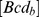	Concentration of site-bound Bcd (or of Bcd-bound sites)
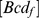	Concentration of free Bcd
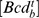	Concentration of site-bound fluorescent Bcd
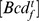	Concentration of free fluorescent Bcd
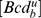	Concentration of site-bound non-fluorescent Bcd
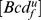	Concentration of free non-fluorescent Bcd
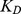	Dissociation constant, 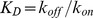 , of scheme (5)
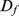	Diffusion coefficient of free Bcd
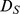	Diffusion coefficient of free and Bcd-bound sites
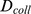	Collective effective diffusion coefficient (Eq. (2))
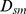	Single molecule effective diffusion coefficient (Eq. (2))

The concentrations listed above satisfy the following relationships: 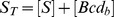, 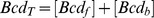, 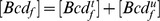, 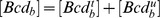. In equilibrium they also satisfy other relationships (see supplementary text 0.2). Since we work with data obtained under different conditions in parts of the text we also use the following superscripts: 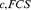 to identify values derived from **FCS** experiments performed in the cytoplasm during interphase (*i.e.* cytoplasmic values during interphase at a position along the embryo that corresponds to the one probed with **FCS** experiments); 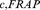 to identify values derived from **FRAP** experiments performed in the cytoplasm during mitosis (*i.e.* cytoplasmic values during mitosis at a position along the embryo that corresponds to the one probed with **FRAP** which we assume is the same as the one probed with **FCS**) and 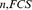 to identify values derived from **FCS** experiments performed in nuclei (again at the location along the embryo that is probed with **FCS**).

### FCS and FRAP yield consistent estimates of Bcd effective diffusion

We first analyze the results derived from **FCS** experiments performed in the cytoplasm during interphase [15]. From the experiments performed using NLS-EGFP we estimate the free Bcd coefficient, 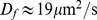. From the experiments performed using Bcd-EGFP we derive the estimates 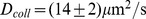, 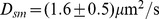, and 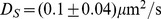 if we use the results of the three component fit of Abu-Arish et al. [15] while we obtain 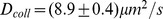, 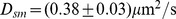 and 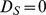 if we use the two component fit instead. Thus, our interpretation of the **FCS** experiments performed in the cytoplasm during interphase is that Bcd-EGFP has a relatively large free diffusion coefficient, 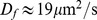, but that inside the embryo it also binds to sites that diffuse very slowly (with 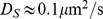 or less). The net Bcd-EGFP transport that results from its free diffusion and binding and unbinding to 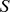 is characterized by two effective diffusion coefficients that differ by an order of magnitude (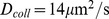 and 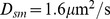 or 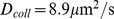 and 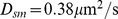 according to the three or two component fit estimates). As we mentioned before, **FRAP** yields the value, 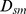. In fact, the value derived for this coefficient from the **FCS** experiments using the three component fit is of the same order of magnitude as the one derived using **FRAP** by Abu-Arish et al. [15] (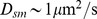) and the one obtained using the two component fit is closer to the result obtained with **FRAP** by Gregor et al. [16] (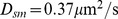). However, we must remember that the **FRAP** and **FCS** experiments that we analyze here were performed during mitosis and interphase, respectively. Thus, we can expect the relevant concentrations and, thus, the effective diffusion coefficient values to be different. Assuming that the free coefficients, 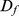 and 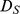, and the dissociation constant, 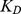, do not change between mitosis and interphase, we conclude that a 20% change of the free binding site concentration can explain a variation of 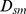 between 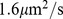 during interphase and 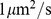 during mitosis (see supplementary text S2).

Our interpretation of the timescales derived from the **FCS** experiments enables us to determine the ratio of concentrations and of 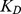 with respect to any concentration in the cytoplasm during interphase at the location where the **FCS** experiments are performed. Using the estimated values of 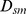, in the cytoplasm during interphase (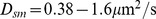 from **FCS** experiments) and during mitosis (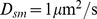 from **FRAP** [15]) and assuming that the ratio of total Bcd concentrations in both situations, 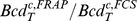, is the same as that of fluorescent Bcd (which we estimate from Ref. [16]), we can also derive the ratios of all the concentrations during mitosis with respect to the total cytoplasmic Bcd concentration during interphase at the location of the **FCS** experiments, 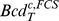. We show in Table 2 the ratios derived using the parameters of the two component fit of Abu-Arish et al.[15] (first value listed in each cell) and the three component fit (second value listed in each cell). In all cases, the values listed were derived from the mean values obtained with the fits.

**Table 2:** Estimates of equilibrium concentrations and of model parameters derived from experiments performed in the cytoplasm.

	Cytoplasm, Interphase	Cytoplasm, Mitosis
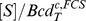	0.02–0.03	0.009–0.05
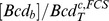	0.98–0.92	1.14
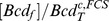	0.02–0.08	0.06
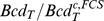	1	1.2
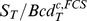	1–0.95	1.15–1.19
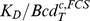	0.00047–0.0026	0.00047–0.0026
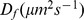	19	19
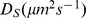	0–0.095	0–0.095
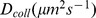	8.9–14	16.7–10.4
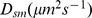	0.38–1.6	1

Parameters derived from fits to **FCS** experiments performed using Bcd-EGFP during interphase in the cytoplasm (first column, using data from [15]) and values estimated for mitosis (second column) assuming that 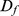, 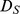 and 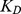 remained invariant and that 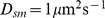 during mitosis (diffusion coefficient estimate derived in. [15] using **FRAP**). The value 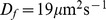 was derived from fits to **FCS** experiments performed using NLS-EGFP. Two sets of **FCS** fits from [15] were used which gave the two limiting values listed in the Table (2 and 3 component fits, respectively). The mean fitting parameters reported in [15] were used to obtain the values listed in the table. All ratios listed are computed with respect to the total cytoplasmic Bcd concentration during interphase at the location where the **FCS** experiments were performed, 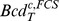.

**Figure 4:**
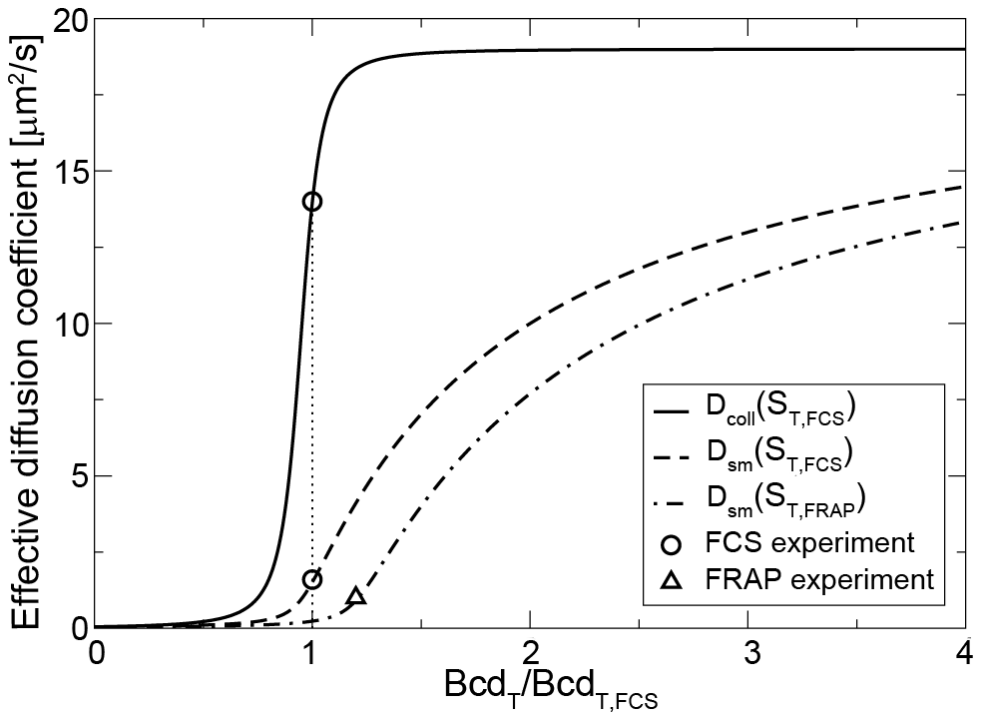
Dependence of the effective diffusion coefficients on the total cytoplasmic Bcd concentration, 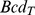
 **, for fixed values of the total concentration of binding sites,** 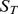 **, and of the dissociation constant,** 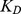 **, as prescribed by our theory.** The solid and dashed curves correspond, respectively, to 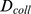 and 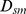 for the estimated value of 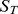 at interphase (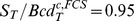 inferred from **FCS** experiments). The dashed-dotted curve corresponds to 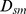 for the estimated value of 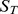 during mitosis (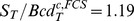 inferred from **FRAP** experiments). We used 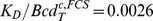 in all cases. The symbols correspond to the situations probed with **FCS** (circles) and **FRAP** (triangle) experiments for which 
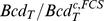
, is equal to 1 and 1.2, respectively.

The concentration 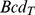 changes along the axis of the embryo. Thus, the concentration ratios, and, consequently, the effective diffusion coefficients, 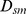, and 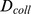, given by Eq. (2) could vary along the axis as well. We do not know what the binding sites are. If we assume that they are uniformly distributed in the cortex along the axis of the embryo as nuclei are and that 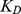 does not vary either then, 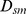, and 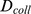, would only change along the axis due to changes in 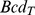. Using Eq. (2) and the relations that the various concentrations satisfy at equilibrium we can rewrite the expressions for 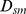 and 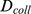 in terms of the ratios 
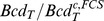
, 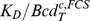 and 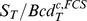 (see supplementary text S2). In particular, setting 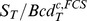 equal to the value derived from the **FCS** or the **FRAP** experiments we can determine how 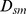 and 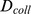 vary with cytoplasmic 
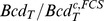
 for the interphase (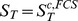) or the mitotic (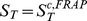) conditions, respectively. We show in Figure 4 plots of 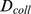 (solid line) and of 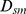 (dashed line) obtained in this way using the total concentration of binding sites derived for interphase (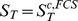) and a plot of 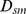 (dashed-dotted line) using the total concentration of binding sites derived for mitosis (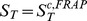). Based on this Figure we conclude that the dissociation constant, concentrations and free diffusion coefficients of the species involved are such that 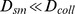 for a wide range of 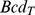 values, which include both the ones probed with **FRAP** and **FCS**. Therefore, it is reasonable that the two techniques report widely different diffusion coefficient estimates.

### Consistency test I: The ratio of nuclear to cytoplasmic total Bcd concentration estimated from FCS fits is within the observed ratios of mature [Bcd-EGFP]

The ratio of the nuclear to cytoplasmic concentrations of mature Bcd-EGFP may be estimated from the data presented by Gregor et al. [16]. It can also be derived from the results of **FCS** experiments under some assumptions. In the absence of detailed experiments, we consider the simple assumption that the dissociation constant of the binding/unbinding processes that Bcd undergoes in the nuclei and in the cytoplasm is the same. In this way we can combine the analyses of the **FCS** experiments performed in the cytoplasm [15] and in the nuclei [20] to obtain the ratio of nuclear to cytoplasmic Bcd concentration. Here we analyze to what extent these two estimations match each other.

Porcher et al. [20] only report the parameters of a 2-component fit to the ACFs obtained using NLS-EGFP and Bcd-EGFP. Working as in the case of the experiments of Abu-Arish et al. [15], we obain the values listed in Table 3 where 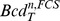 is the total Bcd concentration in nuclei at the location where **FCS** experiments were performed. Again, only the values derived using the mean fitting parameters are listed in the table. In order to check the compatibility of the results of Tables 2 and 3, we assume that the 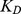 between Bcd and the putative binding sites is the same in the cytoplasm and in nuclei. We then combine the ratio 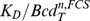 of Table 3 with the value 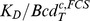 derived from the 2-component fit listed in Table 2 (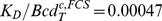) to determine 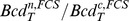. We obtain 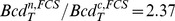, which is within the ratios of mature [Bcd-EGFP] reported by Gregor et al. [16]. This ratio is reduced by a half if we assume that the dissociation constant in the cytoplasm during interphase is twice as large as the one in nuclei.

**Table 3:** Estimates of equilibrium concentrations and of model parameters derived from experiments performed in nuclei.

	Nuclei, Interphase
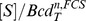	0.018
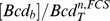	0.989
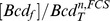	0.011
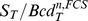	1.007
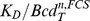	0.0002
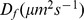	20
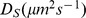	0
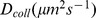	7.7
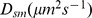	0.22

Parameters derived from fits to **FCS** experiments performed using Bcd-EGFP during interphase in nuclei (using data from [20]). The value 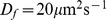 was derived from fits to **FCS** experiments performed using NLS-EGFP. Only the results derived from a 2-component fit were presented in [20]. The mean fitting parameters reported in [20] were used to obtain the values listed in the table.

### Consistency test II: The estimated change in total cytoplasmic binding sites concentration between interphase and mitosis is similar to the observed change in [Bcd-EGFP], which is consistent with a change of available volume

The ratio between fluorescent Bcd-EGFP in mitosis and in the cytoplasm during interphase is of the order of 1.2 (see Fig. 3 in Gregor et al. [16]). Using the values derived from our analysis of the **FCS** and **FRAP** experiments listed in Table 2 we obtain that the equivalent ratio for 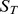 is 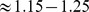. The similarity between both ratios can be interpreted very simply as due to a change in the available volume between interphase and mitosis. This becomes clear in the argument that follows with which we derive a rough estimate of the ratio of available volumes. Let us call 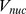 and 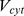 the volume occupied by nuclei and cytoplasm, respectively, during interphase in the region where the **FCS** and **FRAP** experiments are performed. These values change as the divisions proceed, but let us consider they represent some mean value between two consecutive divisions. Because the nuclear membrane disappears during mitosis, the cytoplasmic volume during mitosis is 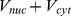. Assuming that the total number of Bcd molecules in the region immediately before and immediately after nuclei division is conserved, we have: 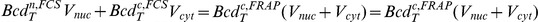. Setting 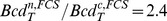 (the value we derive from our analysis of **FCS** experiments in nuclei and the cytoplasm if we assume that the dissociation constant is the same in both cases) and 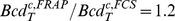 (the value inferred from the figures of Gregor et al. [16]) we obtain 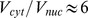. We derive a similar value if we use the total binding sites concentration instead. The volume ratio estimate is reasonable. It implies that the ratio of length-scales, 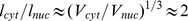, which is consistent with having a spacing between neighboring nuclei of the same order as the nuclei diameters, a very reasonable feature [16].

### From ratios to absolute concentration values

Using the relative weight of the various components of the ACF, the fraction, 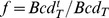 of fluorescent to total Bcd-EGFP molecules can be estimated. However, as described in supplementary text S2, there are some uncertainties regarding the correct expression for the weights. Using different expressions we estimate 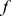 to be between 0.7 and 0.99. The total concentration of fluorescent Bcd, 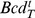, is not very well known either and a wide range of values, 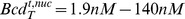, is given in Abu-Arish et al. [15]. The relationship between 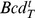 in nuclei and in the cytoplasm on the other hand varies along interphase which adds another uncertainty. We use 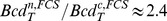, which is among the possible ones, and the rough estimate 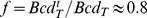 to convert ratios (Tables 2 and 3) to absolute values of concentrations and of the effective dissociation constant between Bcd and its binding sites. In particular, using the same value of 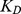 when combined with the 2-component **FCS** fitting parameters obtained in nuclei and in the cytoplasm during interphase, 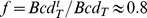, 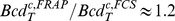 and 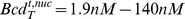, we obtain the values of Table 4. These values, however, should be considered with great care due to all the uncertainties involved in their derivation.

**Table 4:** Absolute values of concentrations and of the binding/unbinding dissociation constant.

	Cytoplasm, Interphase	Cytoplasm, Mitosis	Nuclei, Interphase
Bcd-EGFP (nM)	8–59	9.6–71	19–140
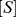 (nM)	0.2–2.2	0.9–4	0.4–3.2
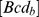 (nM)	9–72	11.4–84.5	23–173
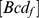 (nM)	0.2–6	0.6–4.5	0.26–2
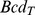 (nM)	10–74	12–89	24–176
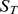 (nM)	9.5–74	11.5–88	24–176
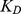 (nM)	0.005–0.2	0.005–0.2	0.005–0.035

Parameters derived from Tables 2 and 3 assuming 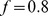 (see supplementary text S2 for details). The smaller range of 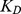 values in nuclei is due to the fact that only data from a 2-component fit to the ACF are presented for this case, while in the cytoplasm the results obtained both for 2 and 3-component fits are presented and this enlarges the range of 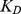 values compatible with the observations.

## Discussion

We have considered a simple biophysical model to analyze the different experiments that have been done to estimate the rate at which Bcd diffuses in *Drosophila* embryos. We have shown that the disparate estimates obtained using **FRAP** [16] and **FCS** [15] are perfectly consistent within this simple model. Namely, they can be explained in terms of two distinct effective diffusion coefficients, 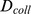 and 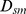 [5]. In our simple biophysical model effective diffusion coefficients describe the net transport that results from the combination of free diffusion and binding/unbinding processes when this transport is observed over a long enough time. The collective diffusion coefficient, 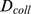, describes the rate at which concentration inhomogeneities spread out with time while the single molecule one, 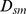, characterizes the distance that an individual molecule travels during a given time. As illustrated by Videos S1 and S2 and Figs. 1 and 2 both coefficients coincide in the absence of the inter-particle coupling that the binding/unbinding processes introduce but otherwise can be arbitrarily different between themselves, with 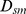 always equal or smaller than 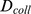. The existence of two different diffusion coefficients, one that describes the mean-square displacement of a molecule and another that gives the rate of decay of a concentration gradient also occurs in crowded, non-ideal solutions, particularly those involving polymers [3], [6], [7]. In our model the interaction between the molecules of Bcd that underlies the existence of the two disparate transport rates is provided by the presence of binding sites with which Bcd interacts. The time after which the net transport can be described by these effective coefficients depends on the relationship between the diffusive and reaction timescales [12]. Once this occurs, **FRAP** experiments give information on the single molecule coefficient [5], [6], [12]–[14] while those that use **FCS** can give information on both [6], [12]. Particle tracking experiments give the single molecule coefficient as well. In **FCS** the autocorrelation function of the observed fluorescence fluctuations is computed and subsequently fitted to determine correlation times, and, from them, diffusion coefficients. In this paper we have analyzed the experimental data of Abu-Arish et al. [15], Gregor et al. [16] and Porcher et al. [20] under the assumption that the timescales are such that the derived transport coefficients correspond to effective ones. One could wonder what conclusions would be drawn if this were not the case. It is under this assumption, however, that we can explain the disparity between the diffusion coefficients estimated using **FCS** and **FRAP**. It has been argued [15] that the disparity could be due to an experimental limitation of **FRAP**. Namely, the recovery time derived from **FRAP** by Abu-Arish et al. [15] is of the same order of magnitude as the time it takes to photobleach the observation volume. This means that once the photobleaching is over and the recovery is monitored there is a noticeable fraction of bleached molecules outside the observation volume. If the data is fitted as if this fraction were negligible the recovery time and, consequently, the diffusion coefficient, are understimated [26]. Numerical simulations of our simple model show that this effect cannot account for over an order of magnitude difference between the coefficients determined using **FRAP** and **FCS** (see supplementary text S2).

Our approach differs from fitting "blindly'' the experimental data since, by using explicit expressions for the correlation times (and the weights) in terms of the parameters of an underlying biophysical model, we can combine observations performed under different experimental conditions and, in this way, estimate free (instead of effective) diffusion coefficients, concentrations and the reaction dissociation constant. According to our analyses, the experiments of Abu-Arish et al. [15] and of Gregor et al. [16] are compatible with Bcd having a free diffusion coefficient 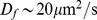 and interacting with immobile or slowly moving sites (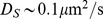). The transport rate of Bcd is then limited by these two values and is larger the larger its concentration. We have also determined that in the region where **FCS** experiments are performed, the majority of Bcd (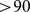
%) is bound to sites and that a similarly large fraction of sites is also bound. This implies that the affinity of Bcd for the binding sites is high. Although Bcd physically interacts with several proteins [22]–[24], it is probably its binding to mRNAs [25] that most significantly affects its diffusion. First, mRNAs are relatively large, and thus diffuse more slowly than proteins. Second, Bcd has already been shown to bind tightly to the homogeneously distributed caudal mRNA. In addition when in the nucleus, it might spend a significant amount of time bound to DNA. Actually, it has been determined that Bcd binds cooperatively to multiple sites of DNA and that this results in a higher affinity (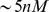) [27]. Our model is very simplified regarding binding. It is implicit in the scheme (5) that the sites act independently of one another. If we replace this scheme by one with cooperative binding we expect the estimated values of 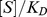 and 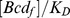 to be smaller than the ones derived using the simple model (5). This, in turn, would imply a larger value of 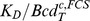 (see supplementary text S2). Thus, the estimate of the relationship between the dissociation constant and the total concentration of Bcd listed in the Tables should be considered as some sort of effective value. A simple scheme like the one in (5) was used in the model introduced by Deng et al. [28] to study the dynamics of the Bcd gradient in Drosophila embryos. According to the analysis presented by these authors the stability of [Bcd] inside nuclei and other properties are dependent on the binding/unbinding equilibrium of Bcd molecules to DNA sites. Deng et al. [28] explore the parameter space of their model under the assumption that 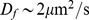 and determine that a 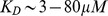 guarantees the stability of the Bcd gradient along division cycles. Their estimate of 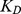, however, depends on the assumed value of 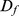. It would have been much smaller if they had assumed 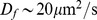, the value that we deduce from our analysis of the experiments of Abu-Arish et al. [15].

**Figure 5:**
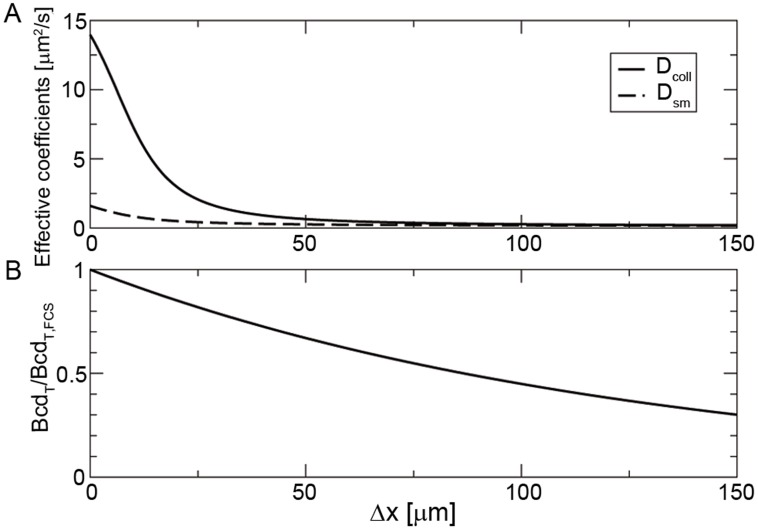
Effective diffusion coefficients and Bicoid concentration as functions of position along the embryo. Top: Effective diffusion coefficients, 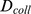 with solid lines, 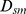 with dashed lines, as functions of the distance to the location where **FCS** and **FRAP** experiments were performed, 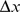, for a uniform concentration of binding sites consistent with its estimated cytoplasmic value during interphase (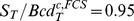) and for a total concentration of Bcd that decreases exponentially with distance with a 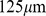 decaying length. Bottom: corresponding exponential profile of total Bcd.

Our interpretation of the experimental results of Abu-Arish et al. [15] and of Gregor et al. [16] can be probed with other experiments. In particular, the non-uniform distribution of Bcd along the embryo implies that the effective coefficients that may be estimated with **FCS** or **FRAP** could, in principle, vary with position too. We can compute by how much they should vary with the distance to the location where **FCS** and **FRAP** experiments are usually performed (the anterior pole) if we assume, as before, that the concentration of binding sites is spatially uniform. In particular, assuming that the total Bcd concentration decays exponentially with a characteristic lengthscale 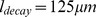 we can go from Figure 4, in which the coefficients are plotted as functions of 
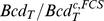
 to a figure in which they are plotted as a function of the distance to the typical **FCS** location, 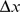. We show the results obtained in Figure 5 where we have plotted 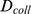 and 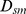 as functions of 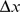. There we observe that at 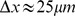, 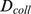, is reduced to about 20% of its value at the anterior pole. Although this numerical estimate is rough, we expect that changes of 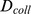 along the embryo should be detectable using **FCS**.

Albeit with its uncertainties, our approach provides a self-consistent picture of a variety of observations. The establishment of the Bcd gradient is a nonlinear process and an accurate estimate of the time it takes to develop should be obtained with a reaction-diffusion model in which diffusion, binding and unbinding are described separately. The actual transport of Bcd is not purely diffusive although it can be characterized by effective diffusion coefficients that are concentration-dependent and vary along a gradient. This means that the transport of Bcd involves a multiscale diffusion process, to some extent, similar to the process analyzed by Daniels et al. [29]. In any case, it is the collective, rather than the single molecule effective coefficient that gives a rough estimate of the (local) rate at which concentration inhomogeneities spread out with time. Within our interpretation of the results of Abu-Arish et al. [15] it is 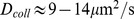. Thus, a gradient over a lengthscale 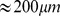 could be established within 48–70 min. Bcd, on the other hand, regulates the expression of various genes and its gradient plays a relevant role since certain proteins are synthesized at very specific locations along it. The very small number of Bcd molecules implies that fluctuations are important. The expression of these downstream genes, however, occurs with high precision. In particular, the analyses of Gregor et al. [30] have estimated this precision at 10%. As discussed by these authors, the physical limit to concentration measurements is determined by the dynamics of molecules arrivals at their targets. This, in turn, is determined by the diffusion coefficient of the molecules. Based on the estimate of this coefficient obtained using **FRAP** [16], the studies of Gregor et al. [30] concluded that the system would need a very long time (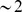 hours) to average out the fluctuations to obtain the observed level of precision. The authors then invoked an average in space to reconcile the estimate of the diffusion coefficient of Gregor et al. [16] and the 10% precision of the read-out mechanism. We must recall that these computations used the estimate of the diffusion coefficient obtained with **FRAP** thus, the single molecule coefficient, 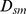. However, it is 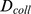, not 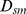, that determines the mean time of separation between subsequent arrivals of the signaling molecules (Bcd) at their targets. According to our estimates, 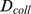 is at least an order of magnitude larger than 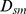 (and it could be twice as large as the one used by Gregor et al. [30]) at the location where the **FCS** experiments were performed. In particular, computing the time, 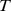, it could take to achieve a 10% precision as done by Gregor et al. [30] before invoking the spatial averaging but with 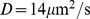 we obtain 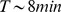 which fits within a nuclear cycle. We must recall, however, that both 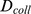 and 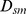 would decrease with the distance to the anterior pole if the concentration of binding sites remains constant along the embryo (see Fig. 5). Thus, it is not certain that the value determined from the **FCS** experiments is the one that should be used. A more detailed study is necessary to address the problem of the read-out mechanism precision.

Our approach involves several simplifications, in particular, the assumption that Bcd interacts non-cooperatively with a single type of binding site. For the analysis of the **FCS** experiments of Abu-Arish et al. [15] we also assume that the system is in a regime such that the net transport of Bcd can be described in terms of effective diffusion coefficients. This is supported by the goodness of the fits presented by Abu-Arish et al. [15], although a picture including anomalous diffusion could also hold. In favor of our model we have shown that it is self-consistent, but because of all its simplifications the numbers we derive might not be completely accurate. More detailed studies are necessary to draw a more definitive picture. In any case we do think that it is the coupling between Bcd molecules that is introduced by the binding with almost immobile sites that can explain the disparate values of Bcd diffusion estimated with **FRAP** and **FCS**, solve the problems associated with the timescale of the Bcd gradient formation and help understand the precision of its read-out mechanisms.

## Materials and Methods

### Analysis of data from FCS experiments

In **FCS** fluorescence fluctuations around equilibrium of a small illuminated volume are measured and their auto-correlation function (ACF) is computed. If the fluctuations are due solely to free diffusion of a single species of fluorescent molecules in and out of the volume, the ACF is of the form: 
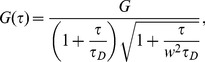
(3)where 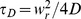 and 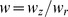. Here 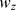 and 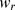 are the sizes of the illuminated volume along the axial and perpendicular directions respectively. In this case, fitting the experimental data to the theoretical ACF Eq. 7 gives an estimate of the diffusion coefficient, 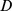. This ideal situation rarely holds in real experiments. In most cases there are multiple components and the best fits are obtained using a superposition of the form:
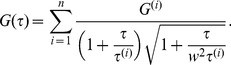
(4)with as many weights, 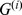, and diffusion coefficients, 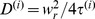, as components (in this case, 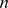). For a system in which there are several freely diffusing non-interacting fluorescent species this superposition has a clear meaning; each component of the ACF gives the free diffusion of a different species and 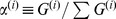 gives the average fraction of fluorescence that the 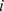-th species contributes to the total fluorescence, 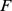, in the volume. Even if, on average, each species contributes with a fixed fraction to 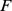, there is no timescale of the problem associated to the mean diffusion coefficient, 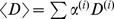. Neither 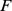 nor any other species diffuses with 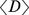. One would be tempted to assume that 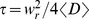 is the characteristic timescale of a particle that diffuses at rate, 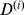, during a fraction of time, 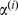, and then changes rate when it binds to another species. However, unless the particles undergo spontaneous inter-conversions (not binding/unbinding with other species), the weighted average, 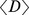 does not set the characteristic timescale of the particle dynamics. As shown next, when the fluorescent species diffuses and binds/unbinds to others and many reactions occur inside the observation volume the fluorescence fluctuation ACF can be written as in Eq. 3 but with some 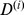
's that are *effective* rather than *free* diffusion coefficients [12]. Thus, the diffusion coefficients that can be extracted from **FCS** experiments are already correctly weighted averages of free diffusion coefficients. As before, the average, 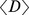, of these already averaged coefficients is not associated to any timescale of the problem.

In this paper we analyze the results of **FCS** experiments performed in *Drosophila melanogaster* embryos that express Bcd-EGFP [16]. More specifically, we use the parameter values derived from the fits to the ACF's presented in [15] and in [20]. They correspond to experiments performed in the anterior cortical cytoplasm during interphase at stage 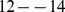 [15] and in anterior nuclei during cycles 13 and 14 [20]. In the case of experiments performed in the cytoplasm several fits are presented in [15] which differ in the number of components of the ACF, among other properties. The best fits correspond to ACF's approximately of the form of Eq. (4) with two or three components (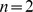 or 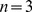 in Eq. (4)) for which the estimated diffusion coefficients and relative weights, 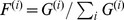, are, for 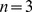: 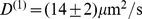, 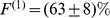, 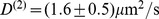, 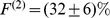, 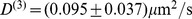, 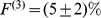, and, for 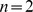: 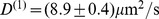, 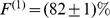, 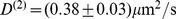, 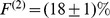. We also use the results obtained in the anterior cortical cytoplasm of embryos expressing NLS-EGFP, a construct with a short nuclear localization signal and a GFP tag identical to that in Bcd-EGFP but that should diffuse freely in the cytoplasm [15]. In this case only the result of a two component fit is presented: 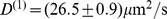, 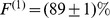, 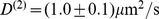, 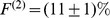. In the case of experiments performed in nuclei only the results of two-component fits are presented both for Bcd-EGFP and NLS-EGFP [20]. The diffusion coefficients and relative weights derived are 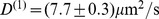, 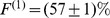, 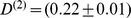 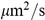, 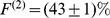 for Bcd-EGFP and 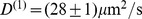, 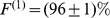, 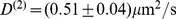, 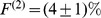, for NLS-EGFP.

### Underlying mechanistic model of Bcd dynamics

We consider the simplest biophysical model that incorporates the presence of binding sites, 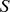, that interact with Bcd-EGFP according to the scheme: 
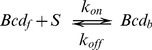
(5)with dissociation constant, 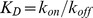. In this equation 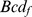 represents the free form of Bcd-EGFP and 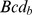 its 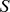-bound form. We assume that both Bcd-EGFP for which GFP is mature (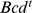) and for which it is not (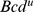) coexist in the system. This assumption is reasonable since it takes several minutes for EGFP to mature and become fluorescent [21], [31]. We also assume that 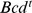 and 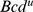 interact with 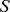 in the same way. The only difference between the tagged (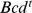) and untagged (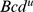) forms of Bcd-EGFP is that GFP is mature for the former (and, thus, fluorescent) while it is not for the latter. Further assuming that the 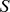 molecules are more massive than the free Bcd-EGFP molecules, so that both 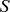 and 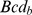 have the same diffusion coefficient, 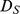, which is in turn smaller than the coefficient of the free Bcd-EGFP molecules, 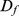, the dynamics of the system is described by the following set of reaction-diffusion equations:



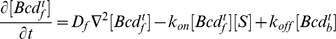


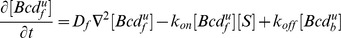


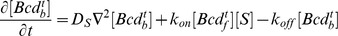


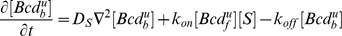


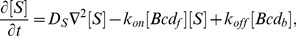
(6)where 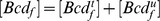 and 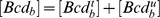. We define 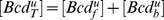 as the total immature Bcd-EGFP concentration (i.e., labeled with immature GFP which is non-fluorescent or untagged) and 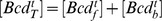 as the total mature Bcd-GFP concentration (i.e., labeled with mature GFP which is fluorescent or tagged). Finally we define 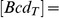 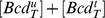 as the total Bcd-EGFP concentration (both fluorescent and non-fluorescent). To analyze the **FCS** experiments we assume that the concentrations are approximately homogeneous within the observation volume and that the mean value of the concentrations are given by the equilibrium condition of the reaction Eq. (5). The first of these assumptions is reasonable since the width of the illuminating spot is 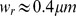 and the typical lengthscale of the Bcd-EGFP gradient is 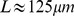. The second one is reasonable as well since the typical timescale of variation of the gradient is much larger than the duration of each **FCS** experiment. Treating fluctuations around this mean as done in [32] we can obtain an analytic approximation to the auto-correlation function (ACF) of the fluorescence fluctuations, 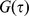, as shown in [12]:



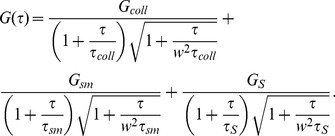
(7)In Eq. (7) 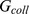, 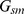, 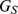, 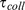, 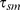 and 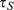 are functions of the biophysical model parameters and concentrations and 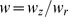 with 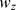 and 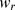 the sizes of the illuminated volume along the axial and perpendicular directions respectively. The times 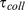, 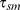 and 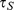 depend on the corresponding (effective) diffusion coefficients as 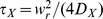 (X  =  coll, sm, S). Thus, the ACF is the sum of three components characterized by three different timescales: one given by the free diffusion of the 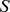 molecules (with weight 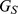) and the other two (with weights 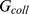 and 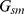) given by the effective diffusion coefficients of Eq. (2). The analytic expression (7) holds as long as the Bcd molecules undergo enough binding/unbinding reactions while they stay inside the observation volume [12], [33]. The weight of the last term becomes 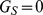 if 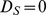 (unpublished data and supplementary text S2). Thus, the ACF reduces to two components in this case.

### FCS experiments and underlying biophysical model

Eq. (7) is formally similar to Eq. (4). The main difference between our formula for 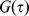 and Eq. (4). is that we have explicit analytic expressions for the weights, 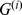, and the times, 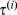, in terms of the variables and parameters of our underlying biophysical model. Therefore, by interpreting the published fitted parameters of [15], [20] in terms of our analytic expressions we estimate values for the parameters and concentrations of our simple mechanistic model for the conditions under which the experiments were done. This interpretation allows us to readily compare the results obtained with experiments, such as the ones done using **FCS** and **FRAP** [16] or **FCS** in the cytoplasm and nuclei, that were performed under different conditions and for which the concentrations could be different. The mapping between our approximation and the fits presented in [15], [20] is done by associating each of the terms in Eq. (7) to one component of the ACF used in [15], [20] according to the relative order of their timescales since for the biophysical model it is 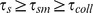. When using the two component fits of [15], [20] we set 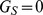 in Eq. (7) and assume 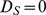. All figures shown in this paper use the results obtained from the three-component fit of [15]. Similar figures are obtained for the two-component fit but with somewhat different numerical values. For comparison purposes, we also derive the parameters of our model in nuclei using the fitting parameters of [20]. Finally, we use the timescales derived from **FCS** experiments performed using NLS-EGFP to estimate the free diffusion coefficient of Bcd-EGF, 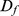. Namely, given that the weights of the two components obtained from the fits of NLS-EGFP experiments satisfy 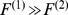 we assume that 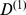 is the free diffusion coefficient of NLS-EGFP from which we derive the free coefficient of Bcd-EGFP considering the different molecular weights of both molecules. This assumption seems to be correct given that the range of values derived in the cytoplasm (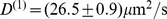) and in nuclei (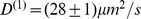) overlap. This does not happen for the experiments performed with Bcd-EGFP, which is an indication that the coefficients derived in this case are effective (concentration dependent) rather than free.

### Particle simulations

Eqs. (6) describe the spatio-temporal dynamics of the concentrations of three species that diffuse and react. This is actually a mean-field description. However it is the individual molecules of the species the ones that move in the medium and eventually react with other molecules when they become close enough. In this paper we present the results of numerical simulations in which we follow the dynamics of the individual molecules as they diffuse in a medium and react according to Eq. (5). In these simulations the binding sites are immobile, *i.e.* they have 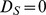. In what follows we will refer to the moving molecules as walkers or free particles and to the particles bound to sites as bound particles. Particles are equivalent to Bcd molecules in the biophysical model of Eqs. (6). Our simulations can be summarized in the following pseudo-code:

1. react.
2. diffuse free particles.
3. increment time by dt and go to 1.

#### Reaction

Each particle is referred to by an index, and similarly each binding site. The particles are either free or bound and the binding sites are either occupied or unoccupied. Each particle and each binding site have coordinates. xyz is the list of particle coordinates. xyztraps is the list of binding site coordinates. "bound" and "occupied" are the lists of bound particles and occupied binding sites, respectively. These two lists are updated at each time step as described below. Pseudo-code for the call to ChemistryUpdate is: 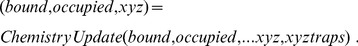
(8)


At each time step we partition space into disjoint boxes and update the lists for each box independently. In what follows we describe how the updates are performed within a given box.

To determine the number of bindings that take place with a box we use the notion of a "Wiener Sausage" which is the volume [34] traced out by a spherical Brownian particle of radius 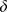 and diffusion coefficient 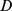 in time 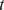: 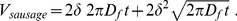
(9)


The survival probability 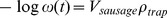 where 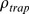 is the number density of the binding sites. The probability that a single walker in a box of volume 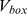 is trapped by a single binding site, 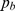, is given by: 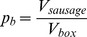
(10)where 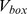 is a volume which we took to be 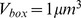 in our simulations. We assume that 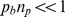 where 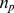 is the number of walkers in 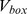. If this condition failed the simulation was aborted and a new realization was attempted. The number of binding reactions for 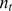 binding sites is obtained by drawing a random sample, 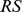, from the multinomial distribution 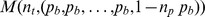 where the vector of probabilities 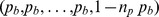 has 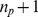 components. The number of bindings, 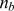, to occur is then the number of nonzero entries in the first 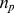 components of the random variable 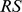 (plus 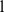 if the last component of 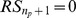). We parametrize the reaction by 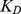 so that the probability of a particle unbinding, 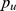 during the interval 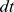 is given by: 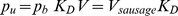. This yields an on-rate of 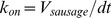 and an off-rate of 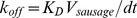. To perform the unbindings we determine how many of the initially bound particles will remain bound,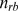, by drawing from a binomial distribution (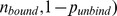. Note that the number of occupied binding sites and bound particles is the same by definition so the number of occupied binding sites that will remain occupied is also 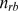. At the end of the time step the updated list of bound particles consists of the union of those previously bound particles that remained bound with the list of those previously free particles that were bound during the time step. Similarly, the updated list of occupied binding sites consists of the union of the previously occupied binding sites that remained occupied with the list of those previously unoccupied binding sites that were occupied during the time step. Particles that are selected to bind to a binding site are moved to the location of their reaction partner (*i.e.* the binding site they bound to). We neglect fluctuations in the sausage volume and thus cannot claim that these simulations are quantitatively accurate but they are adequate to illustrate the subject at hand.

#### Diffusion

First the list of free particles is obtained by taking the complement of the list of all particles with respect to the bound particles. The 3 spatial coordinates of each free particle is incremented by drawing 3 zero mean normally distributed random variables with variance 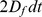 and adding them to the current position. Then each particle is checked to see if it is still in the simulation volume which we take to be 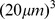. If any coordinate of any particle is outside the volume by a distance 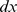 that particle coordinate is reflected back across the boundary. For example let 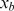 be the location of a boundary in the 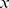 direction. If, after a diffusive step, the 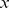-coordinate of a particle exceeds 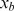 by 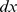 so that 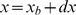 is outside the simulation volume then the reflected coordinate is 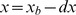. This is done for all 3 spatial coordinates.

#### Sets of particle simulations and parameters

In this article we perform three sets of particle simulations: (1)Free particles in the absence of binding sites, (2)Simulated **FRAP** -like experiment and (3)Simulated particle bolus experiment. In the last two sets of simulations binding sites are present and free particles react with them according to Eq. (1). In all cases, the volume simulated is a cube 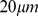 on a side and the free diffusion coefficient of the walkers is 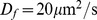. Both in the free particle (no chemistry) simulations and in those of the particle bolus experiment a bolus of free fluorescent particles is initially added to a non-fluorescent pre-existing equilibrium inside the central 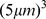 cube. To make these two simulations comparable the number of added particles is 1,875 in both simulations while the total number of particles in the pre-existing equilibrium inside the simulation volume is 20,000 in the absence of binding sites and 600,000 when binding sites are present. Given that in the simulations we follow the individual particles we can do statistics over all the particles or over the added (fluorescent) ones. We do so as explained later. In the **FRAP** -like experiment an initial equilibrium situation is assumed but with all free and bound particles being fluorescent. At 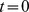 the free and bound particles inside a spherical volume, 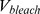, of radius 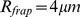, are bleached. The equilibrium condition in the **FRAP** -like experiment is the same as in the simulated bolus experiment with binding sites. The reaction rates and diffusion coefficients also coincide.

The parameters of the simulations with binding sites are: 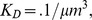


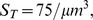


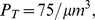


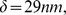


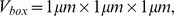
where 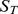 and 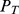 are the total concentrations of binding sites and of particles, respectively, before the addition of the fluorescent particles in the bolus simulation.

#### Simulation diagnostics

For all three simulation sets we compute the mean square displacement (**MSD**), 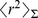, as: 
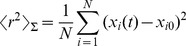
(11)where the subscript, 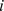, refers to each individual particle for which the mean square displacement is computed, 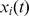 and 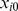 are the 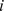-th particle positions (in three space dimensions) at time, 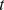, and at the initial time and 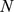 is the total number of particles over which the sum is performed. In the simulations with added particles we perform this computation over the fluorescent particles (*i.e.* over the particles that were initially added to the pre-existing equilibrium). In the **FRAP** -like experiment, we do it for the particles that are initially bleached.

For the simulations with added particles we also compute the second moment of the particle distribution (which, in certain circumstances, can be interpreted as a distributional **MSD**). This involves performing a numerical version of an integral of the form 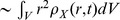 over the simulation volume where 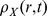 is the concentration of particles of type 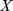 at position 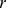 and time 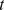. Here 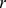 is the three-dimensional position measured from the center of the simulation volume. We do this both for the fluorescent (*i.e.*, added) particles and for all of them. In both cases we approximate the integrals by partitioning space into boxes and counting the particles in each box. We denote the number of particles in the 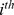 box by 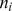. The squared distance of the geometric center of the 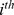 box from the origin is denoted 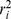. Then we approximate 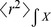 by: 
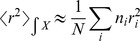
(12)where 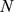 is the number of added particles. When 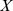 refers to the fluorescent (*i.e.* added) particles, 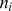 is the number of fluorescent particles in the 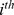 box at each time. We use the subscript 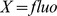 to identify this case. When 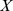 refers to both the fluorescent and non-fluorescent (*i.e.* all) particles 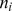 is the number of all particles in the 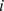-th box. We use the subscript 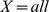 to identify this case. It is 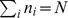 for 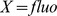 while 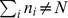 when 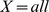. As explained in the supplementary text S3, in the long time limit, 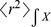 scales linearly with time according to:
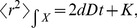
(13)with 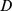 a diffusion coefficient, both for 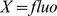 and 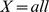. In the absence of binding sites it is 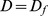 in all cases. If there are binding sites, 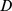 is different depending on whether 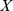 refers to the fluorescent particles or to all of them. As we show with the simulations, it is 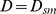 in the former and 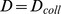 in the latter with 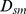 and 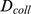 defined in Eq. (2).

For the **FRAP** -like experiment we also compute the "relative fluorescence'' inside the initially bleached volume, 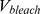 as: 
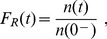
(14)where 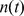 is the number of fluorescent molecules in the bleached volume at time 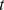 and 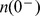 is the number of fluorescent particles in the bleached volume just before the bleaching took place.

## Supporting Information

Text S1 **Diffusion, length and time scales.** In this text we give a brief introduction to normal, anomalous and effective diffusion.
(PDF)

Text S2 **Detailed description of the mapping between model and experiments.** In this text we discuss the main assumptions that underlie the application of our simple biophysical model to interpret the results obtained from **FCS** and **FRAP** experiments performed in *Drosophila* embryos that express Bcd-EGFP. More specifically we compare different possible mappings, discuss the compatibility of the models assumptions with the observations, their limitations and possible extensions.
(PDF)

Text S3
**Mean square displacement and second moments of particle distributions.** Here we describe the meaning of the various diagnostics that we perform on the particle simulations. In particular, we show how the slope of the **MSD** and of the different second moments, 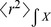, that we compute are related to the different diffusion coefficients that we discuss in this paper.(PDF)

Video S1
**Free particle diffusion.** This video shows the results of a simulation of a system of freely diffusing particles with 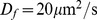. In this simulation a bolus of 1,875 fluorescent particles is added to the central 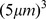 cube in a background of 20,000 particles that are uniformly distributed over the 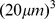 cubic simulation volume (see Materials and Methods for details). The video has three panels. In each of them we project the 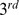 dimension into the plane that is shown. The left-most movie shows the local deviation in concentration of all particles above the equilibrium concentration. The fluctuations in the equilibrium baseline are apparent near the borders. The center movie shows the concentration of the added (fluorescent) particles. In this case the concentration of added particles near the borders begins at zero so the fluctuations of this quantity in the early part of the movie are small near the borders. As time passes and the particles spread those fluctuations grow. The right-most movie shows the actual (2D projection of the) positions of the added particles. We quantify the rate of spread of the distributions in the left and center panels by means of the second moments, 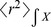, (Eq. (12)) computed using all the particles (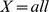) and only the (fluorescent) added ones (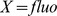), respectively. Both second moments grow linearly with time with the same slope as shown in Figs. 1 A and 1 B. This slope coincides with that of the averaged mean square displacement of the individual particles Fig. 1 C. From the slopes we obtain 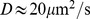 which agrees, in turn, with the diffusion coefficient of the particles that was used in the simulation. In the case of free diffusion the rate at which a perturbation spreads out with time and at which the mean square displacement of the individual particles increases is ruled by the same diffusion coefficient.(MOV)

Video S2
**Effective diffusion.** This video shows a simulated experiment in which a bolus of fluorescent particles is added to the central 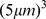 cube in a 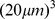 cube in which particles diffuse and react with immobile binding sites according to Eq. (1). Particles and sites are initially uniformly distributed and at chemical equilibrium (see Materials and Methods for details). As in Video S1 the left-most panel shows the concentration of all the particles above equilibrium. The center panel shows the concentration of the added (fluorescent) particles. The right-most panel shows (a 2-dimensional projection of the) positions of the added particles. This is what would be observed if each of the added particles could be identified. The deviation from equilibrium of the concentration of all the particles smooths out so fast that is only obvious in the earliest frames of the left most panel. This smoothing occurs much faster, on the other hand, than that of the deviations in the fluorescent particle density. This difference becomes quantifiable in Fig. 2 where we show the second moments 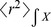 (Eq. (12)) computed using all the particles (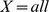) in A and only the (fluorescent) added ones (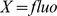) in B. In both cases the second moments eventually depend linearly on time. From the slopes we obtain a diffusion coefficient that is more than 10 times faster in Fig. 2 A than in Fig. 2 B. The latter, on the other hand, is roughly the same as the one that is derived from the slope of the mean square displacement shown in Fig. 2 C. These observations agree with the results of [5] (see also supplementary text S3). Namely, according to the theory, the deviation from equilibrium of the total particle concentration spreads with the collective diffusion coefficient, 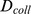, and that of the (fluorescent) added particles with the single molecule coefficient, 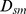, which also rules the time dependence of the individual particles mean square displacement. For the simulation parameters, it is 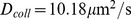 about 14 times faster than the single molecule diffusion coefficient, 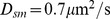, which agrees with what is observed in the panels and in Fig. 2.(MOV)

Video S3
**FRAP.** This video shows a simulated **FRAP**-like experiment for a system like the one probed in Video S2. In this simulation all the particles (free and bound) are assumed to be initially fluorescent and at equilibrium with the binding sites. The chemical parameters and rates are the same as in Video S2 (see Materials and Methods for more details). At 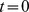, the particles in a spherical volume (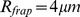) which is in the center of the 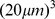 simulation volume are bleached. The simulated particles are diffusing in 3 dimensions but only two coordinates are shown. In this video we show all of the bleached particles (BLUE) and those unbleached particles (RED) that are inside the bleached volume. We observe how the fluorescence in the bleached volume recovers with time due to the diffusion of the free fluorescent particles. This recovery is quantified in Fig. 3.(MOV)
