# Supplementary material for: Messages Do Diffuse Faster than Messengers: Reconciling Disparate Estimates of the Morphogen Bicoid Diffusion Coefficient
Source: PLoS Comput Biol. 2014 Jun 5;10(6):e1003629. doi: 10.1371/journal.pcbi.1003629 (PMC4046929; doi:10.1371/journal.pcbi.1003629)
Supplement: Text S1 — Diffusion, length and time scales. In this text we give a brief introduction to normal, anomalous and effective diffusion. (PDF) [file pcbi.1003629.s001.pdf]

Text S1: Diffusion, length and time-scales

I. FREE DIFFUSION

Particles in solution move erratically due to the continuous collisions with solvent molecules. In the case of a simple solvent this erratic movement is referred to as *Brownian motion* [1]. The first theoretical description of this behavior was presented by Einstein[2, 3]. Einstein's analysis is based on the assumption that the particle "forgets" its direction of motion after a certain time and that the mean distance it travels during this time is finite. Thus, there is a finite time interval, τ, and the direction of motion during each τ-interval is independent of one another [4]. There is a fundamental mathematical result, the *central limit theorem*, that states that the sum of many independent random variables with finite variance is a Gaussian-distributed random variable (see *e.g.* [5]). Thus, since the displacement of the Brownian random walker after a time $t \gg \tau$, $\mathbf{r}(t)$, is the sum of many independent (random) displacements (those performed during the intervals, τ), the probability that $\mathbf{r}(t)$ be within a volume of size d^3r around the value $\mathbf{r}$ is Gaussian-distributed:

$$P(\mathbf{r},t)d^3r = \frac{1}{(4\pi Dt)^{d/2}} \exp(-\frac{\mathbf{r}^2}{4Dt})d^3r. \quad (1)$$

In Eq. (1) d is the number of dimensions of the space over which the particle travels (*e.g.*, $d = 2$ if it is a surface or $d = 3$ if it is a volume) and D is the so called diffusion coefficient which is related to the variance of the independent displacements. In the simple random walker picture in which the mean time between collisions is τ and the mean length traversed during such time is λ, it is $D = \lambda^2/(2\tau)$. The probability density given by Eq. (1) allows the calculation of all the statistical properties of the particle displacement. In particular, one obtains:

$$\langle \mathbf{r}(t) \rangle = 0, \quad (2)$$
$$\langle |\mathbf{r}(t)|^2 \rangle = 2dDt. \quad (3)$$

This implies that the mean square displacement of the particle after a time t, $\langle |\mathbf{r}(t)|^2 \rangle$, grows linearly with the elapsed time. This is characteristic of a normal diffusive behavior. If one considers many particles that perform their random walks independently of one another, then, the probability P given by Eq. (1) also serves to describe the concentration of these Brownian random walkers as a function of position and time, $c(\mathbf{r}, t)$, for an initial condition in which all the particles are located at the origin (so that the displacement after a time t of each of them is equal to its position). Namely,

$$c(\mathbf{r},t) = NP(\mathbf{r},t) = \frac{N}{(4\pi Dt)^{d/2}} \exp(-\frac{\mathbf{r}^2}{4Dt}), \quad (4)$$

with N the total number of particles. This concentration is in fact a solution of the diffusion equation:

$$\frac{\partial c}{\partial t} = D\nabla^2 c. \quad (5)$$

This equation was introduced in 1855 by the German physiologist Adolf Fick in a phenomenological way to describe how water and nutrients travel through membranes. It is a macroscopic equation that involves a certain degree of coarse-graining. Namely, it provides a law for the dynamics of the local changes in the mean number of particles (or, equivalently, its concentration) inside a region that is macroscopically small but microscopically large. In one space dimension, for example, it is $\nabla^2 c = \partial^2 c/\partial x^2$. If one thinks of the spatial domain as divided in macroscopically small regions of size Δx, then $\partial^2 c(x,t)/\partial x^2 \approx ((c(x+\Delta x,t) - c(x,t)) - (c(x,t) - c(x - \Delta x,t)))/\Delta x^2$. Thus, according to Eq. (5), $c(x,t)$ will change in time if the concentrations in the surrounding regions ($x+\Delta x$ and $x-\Delta x$) are different from it. Eq. (5) is a transport equation, it describes "local" concentration changes due to particles movement in or out of the macroscopically small (*i.e.*, local) region. The second derivative in this type of equation is characteristic of diffusive transport in which there is not a mean velocity (see Eq. (2)): the individual particles move at random with equal probability of stepping to the right or the left (in one space dimension) after bumping onto a solvent molecule. The solution of Eq. (5) given by $c(\mathbf{r},t) = NP(\mathbf{r},t)$ with $P(\mathbf{r},t)$ as in Eq. (1) shows that the diffusion coefficient, D, can in principle be estimated from the mean square displacement of a single particle (Eq. (3)) or from the spread of a population of particles whose concentration evolves according to Eq. (4). This is illustrated in Video S1 and in Fig. 1 of the accompanying manuscript where we present the results of a particle simulation of molecules that move randomly in a $(20\ \mu m)^3$ cube with diffusion coefficient $D = 20\mu m^2/s$. In the example a bolus of 1875 "fluorescent" particles is added to the central $(5\mu m)^3$ cube in a background of 20,000 particles that are uniformly distributed in the simulation volume. Since the diffusion equation is linear, the time evolution of the concentration of all the particles or of the deviation of this concentration with respect to the initial equilibrium condition is ruled by Eq. (5). In this example, the deviation with respect to equilibrium is the same as the concentration of added particles. The total concentration perturbation and the added particles concentration depicted, respectively, in the left and middle panels of Video S1, spread out with time at the same rate. This becomes clearer in Figs. 1A and 1B where we can observe that the quantity $\langle r^2 \rangle$ (computed using these two concentrations as described in Materials and Methods) depends linearly with time with the same slope in both cases. As explained in supplementary text S3 the slope of the $\langle r^2 \rangle$ *vs* t curve is

$2dD$ with $d = 3$ the number of dimensions of the simulation (notice that the Videos correspond to projections on the $z = 0$ plane of a three-dimensional simulation). $2dD$ is also the constant of proportionality between the mean square displacement of each added particle (averaged over all of them) and time as shown in Fig. 1C. In fact, this is reproduced by the simulations. We obtain $D = 19.3$, 20.3 and $20.2 \mu m^2/s$ from the curves in Figs. 1A, 1B and 1C, respectively.

II. ANOMALOUS DIFFUSION

This first discussion shows that diffusion involves a coarse-grained description. Namely, it does not hold at timescales at which the particles have a well-defined velocity ($\leq \tau$). In most applications the limiting lengthscale is the mean-free path which is usually a fraction of the molecule diameter [6]. It also shows that diffusion can either be described in terms of the behavior of a single particle or of a collection of particles (*i.e.*, by means of their concentration). It finally shows that in the case of "free", normal, diffusion, the ("long") time dependence of a single particle mean square displacement (Eq. (3)) or the rate at which the concentration of the particle species spreads out with time (Eq. (4)) is determined by the same (free) diffusion coefficient, D. However, in the crowded environment of a cell's interior, the transport of molecules hardly ever corresponds to free diffusion in which the dynamics of the molecules is solely determined by their collisions with a simple solvent [7]. The existence of obstacles or of other interactions, in principle, can give rise to what is called *anomalous diffusion* a situation in which the mean square displacement of a particle grows with time as $\sim t^\gamma$ with $\gamma \neq 1$. In fact, subdiffusive transport (*i.e.* with $\gamma < 1$) has been observed experimentally in porous systems and on cell membranes [6]. Now, the linear scaling between $\langle |\mathbf{r}|^2 \rangle$ and t of normal diffusion is based on a mathematical theorem. Thus, in order for the mean square displacement to have a dependence other than linear with respect to time some of the assumptions of the theorem must not hold. In particular, subdiffusion is obtained with random walks in which there is not a well-defined mean time, τ, between collisions, but rather those times are taken from a long-tailed distribution [8]. This type of behavior can be due to the presence of certain type of "traps" that hold the particles for a while or restrict their movement [9, 10]. In many occasions, however, the anomalous transport only holds within a certain time window. In those cases, if one computes the mean square displacement of a particle after a long enough time (*i.e.* for enough averaging over the individual random steps of the particle of interest) the behavior of the linear dependence between the mean-square displacement and the elapsed time predicted by the central-limit theorem is recovered [1]. The "diffusion coefficient" in this long time limit, however, is smaller than the one that is obtained in the absence of traps or movement restrictions. This discussion shows that binding can lead to a type of transport that is transiently anomalous but that eventually recovers the properties of normal diffusion.

III. EFFECTIVE DIFFUSION

Binding/unbinding corresponds to a chemical reaction. The dynamics of a system in which particles perform a "normal" random walk due to non-reactive collisions with molecules of a simple solvent and undergo binding/unbinding with other molecules can be described by reaction-diffusion equations. For example, in the case of a single species, P_f, that reacts with binding sites, S, to form a complex P_b, according to

$$P_f + S \underset{k_{off}}{\overset{k_{on}}{\rightleftarrows}} P_b, \tag{6}$$

the reaction-diffusion equations are:

$$\begin{aligned} \frac{\partial [P_f]}{\partial t} &= D_f \nabla^2 [P_f] - k_{on} [P_f] [S] + k_{off} [P_b] \\ \frac{\partial [P_b]}{\partial t} &= D_S \nabla^2 [P_b] + k_{on} [P_f] [S] - k_{off} [P_b] \\ \frac{\partial [S]}{\partial t} &= D_S \nabla^2 [S] - k_{on} [P_f] [S] + k_{off} [P_b], \end{aligned} \tag{7}$$

if P_f diffuses with coefficient D_f due to their collisions with solvent molecules and P_b and S do it with coefficient D_S. It is implicit in the latter that S is much more massive than P_f so that binding with P_f does not change its free diffusion coefficient. Eqs. (7) are macroscopic equations that can be derived from a more detailed description of the particles dynamics (see *e.g.* [5, 6, 11]). As with Eq. (5) they involve a degree of coarse-graining and describe the average behavior of the individual molecules of the participating species. It is clear from Eqs. (7) that they model a situation in which "local" changes in the concentrations occur because particles enter and leave the macroscopically small region as they bump onto solvent molecules (represented by the terms $\propto \nabla^2$ as in Eq. (5)) and due to their chemical transformations into one another. We call D_f and D_S *free* diffusion coefficients because they are due to the non-reactive collisions of the corresponding species with the solvent. Concentration changes due to chemical reactions are modeled in Eqs. (7) in the usual way. This implies that there is a probability per unit time that the binding occurs once the two molecules are close enough (in the example of (6), k_{on}/d_i^3 with d_i a typical distance within which the molecules can interact and react) and that the time for unbinding follows an exponential distribution (in the example of (6) of mean $1/k_{off}$). As in the case of pure (free) diffusion (Eq. (5)), the macroscopic description holds for timescales that are much larger than the characteristic time, τ, between non-reactive collisions. The dynamics

prescribed by reaction-diffusion equations is not purely diffusive and, thus, local inhomogeneities in the concentrations do not spread out following Eq. (4). Furthermore, the reactions that lead to Eqs. (7) are nonlinear in the concentrations and this introduces important differences with respect to the linear Eq. (5).

As in the case of free diffusion in which particles only collide non-reactively with the molecules of a simple solvent, when particles also bind or react, two types of descriptions are possible. One may look at how the various species concentrations change with time, as done when using Eqs. (7). One may also "follow" an individual particle as it moves around bumping onto solvent molecules and binding/unbinding to/from other molecules or sites and then compute some statistical quantities such as the particle mean square displacement. Of particular interest is the situation in which this analysis is done when the system is in equilibrium. This is the case of the experiments that we are interested in. In particular, the situations probed by the experiments that we analyze in the accompanying paper are such that the concentrations are approximately uniform, time-independent and in chemical equilibrium with one another inside the region of interest during the observation time. This does not mean that there is no movement. The equilibrium refers to the average behavior of the species populations. One can then still follow an individual particle as it moves around or one can analyze how a small local change in the concentration (a fluctuation) spreads out with time. Even in a case in which diffusion in a simple solvent and reactions are involved, the long time behavior of the mean-square displacement of an individual particle or of a perturbation in the concentration have the same time-dependence as the ones encountered when the molecules only suffer non-reactive collisions with a simple solvent, *i.e.* in the purely diffusive (normal) case characterized by Eq. (3) and Eq. (4). The time after which these relationships hold depends both on the (free) diffusion coefficients and on the reaction rates. The diffusion coefficients that enter the relationships, on the other hand, do not correspond to any of the free diffusion coefficients of the species involved (D_f or D_S in Eqs. (7)) but are weighted averages of them that depend on the species concentrations. We call them "effective" diffusion coefficients. Due to the nonlinearity of the equations, the effective coefficient that rules the time-dependence of the mean-square displacement is different from the one that determines the rate at which local perturbations in the species concentrations spread out with time [12]. We call them single molecule (D_{sm}) and collective (D_{coll}), respectively, and for the simple model described by Eqs. (7) they are given by:

$$D_{coll} = \frac{D_f + \frac{[S]^2}{K_D S_T} D_S}{1 + \frac{[S]^2}{K_D S_T}}, D_{sm} = \frac{D_f + \frac{[S]}{K_D} D_S}{1 + \frac{[S]}{K_D}}, \quad (8)$$

In the case analyzed in the accompanying paper, as in most cases, the binding sites (S) are either immobile or have a very small free diffusion coefficient (D_S) compared to that of the free particles (D_f) because they are assumed to be more massive. In such a case it is $D_S \leq D_{sm} \leq D_{coll} \leq D_f$. Distinguishing between D_{sm} and D_{coll} is important because, depending on the values of the free diffusion coefficients, concentrations and reaction rates, the ratio D_{coll}/D_{sm} can be arbitrarily larger than one [12].

The weighted average that gives D_{sm} has a simple intuitive explanation. Let us assume that we follow an individual particle that bumps into the molecules of a simple solvent diffusing with coefficient, D_f, while it is free, that binds/unbinds to sites, S, according to the scheme (6) in a medium in which the concentration of sites is $[S]$ and that, when bound to S, it also bumps into the solvent molecules but with the diffusion coefficient of the sites, D_S. In view of the scheme (6) we expect that, on average, it takes a time $1/(k_{on}[S])$ for the particle in its free form to bind to a site and a time $1/k_{off}$ for the particle in its site-bound form to become free. This means that, when observed during a long enough time, t, we can expect the particle to spend, on average, a fraction of time $t_f/t = 1/(k_{on}[S])/(1/(k_{on}[S] + 1/k_{off})$ in its free form (and, equivalently, a fraction $t_b/t = 1/(k_{off})/(1/(k_{on}[S] + 1/k_{off})$ in its site-bound form). We can then compute its effective diffusion coefficient as $(t_f/t)D_f + (t_b/t)D_S$. This gives exactly the single-molecule effective diffusion coefficient, D_{sm}, defined in (8). Why do small fluctuations in the particles concentrations eventually spread out with a different effective coefficient? Mathematically, this can be traced back to the nonlinearity of the chemical reaction. In particular, if instead of binding to sites the particles suffer spontaneous transformations between two states ("free" and "bound") characterized by two free diffusion coefficients, D_f and D_S, then both the eventual diffusive behavior of the mean square displacement of an individual particle and the rate of spreading of a concentration perturbation is ruled by $D_{sm} = (t_f/t)D_f + (t_b/t)D_S$ with t_f/t and t_b/t the fractions of time that each particle is free or bound, respectively. The chemical reaction introduces a coupling between the individual particles which is mediated by the sites. Thus, what happens to a collection of particles is not simply the "sum" of what happens to each of them. Let us illustrate the nonlinearity with an example in one space dimension to make the discussion simpler. Consider a pipe with a solution at equilibrium inside it. As we mentioned before, diffusion involves a certain degree of coarse graining. So, let us imagine dividing the pipe into segments of length, Δx, and let us focus on a segment whose center is located at a particular position, x. Let us compare two situations, one in which the (solute) particles diffuse freely and one in which they also bind/unbind to *immobile* traps. In both cases, particles are continuously moving in and out of the segment centered at $x = 0$. How many particles leave or enter it during a certain time interval, Δt, depends on how many (free) particles there are in the neighboring seg-

ments. Since everything is in equilibrium the segment at $x = 0$ receives, on average, during Δt, the same number of particles from the left as from the right. Similar numbers leave the segment at $x = 0$ to the left and to the right during Δt and the mean number of particles it contains remains approximately constant. Suppose that some particles are added to the segment whose center is located at $x = -\Delta x$ and consider what happens in the segment centered at $x = 0$ a time Δt after the addition of the particles. Assume that Δx and Δt are such that the free particles can diffuse over a distance Δx during Δt. Suppose further that we color the added particles but that, otherwise, they behave in the same way as those that there were already inside the pipe. The addition of particles alters the equilibrium situation in such a way that, during Δt, the segment at $x = 0$ receives more particles from the left than from the right and the total number of particles it receives is larger than the number that leave it. Thus, during Δt, there is a net flux of particles as a result of which the segment at $x = 0$ ends up having a surplus of free particles. In the case in which the particles are free all the time, the fraction of all the particles that enter the segment at $x = 0$ from the left during Δt that are colored is the same as the fraction of colored particles that were in the segment at $x = -\Delta x$ just after they were added. Thus, the constant of proportionality between the flux of particles from left to right and the number of particles at $x = -\Delta x$ is the same regardless of whether they are colored or not. In this case there is a unique diffusion coefficient, the free coefficient of the particles. In the case in which particles bind to sites, some of the added (colored) particles become bound during Δt. This additional binding is compensated by a release of some of the site-bound particles. Most likely, the particles that are released at $x = -\Delta x$ during Δt are those that were already bound when the free colored particles were added, *i.e.* they are not colored. It is then likely that the fraction of colored to non-colored particles that are bound in the segment at $-\Delta x$ during Δt be larger than the equivalent fraction of free particles. Thus, while the number of free particles that enter the segment at $x = 0$ during Δt will be proportional to the mean number of free particles that are inside the segment at $-\Delta x$ during Δt, the fraction that enter which are colored will be smaller than the fraction of colored particles that remain in the segment at $-\Delta x$ (because they became trapped). Thus, the constant of proportionality between the flux of particles from left to right and the number of particles at $x = -\Delta x$ is smaller for colored particles than if we consider all of them without distinguishing their color. In this case there are two "effective" diffusion coefficients. If we take this example to the limit of having added only one (colored) particle we arrive at the conclusion that the time it takes for an individual particle to diffuse from $-\Delta x$ to $x = 0$ is different than the rate at which the concentration difference between $-\Delta x$ and $x = 0$ decays by diffusion: while the first one is characterized by the single-molecule diffusion coefficient, D_{sm} the second is determined by the collective diffusion coefficient, D_{coll}. We illustrate the meaning of these two effective diffusion coefficients in Video S2 and Fig. 2. This video and its corresponding figure are equivalent to Videos S1 and Fig. 1 but for a system of particles that diffuse freely with $D_f = 20\mu^2/s$ and react with immobile sites according to scheme (6) (see Materials and Methods for simulation details). The left most panel in Video S2 depicts the deviation of the concentration of all the particles with respect to equilibrium, regardless of whether they are fluorescent or not while the middle one shows the concentration of added (*i.e.* fluorescent) particles. We can observe that the former spreads out faster with time than the latter. This becomes clearer in Figs. 2A and 2B of the accompanying paper where we can observe that the quantity $\langle r^2 \rangle$ (computed using these two concentrations as described in Materials and Methods) changes faster in A than in B. This is due to the effect that we have described in the one-dimensional example: the perturbation of the colored particles diffuses more slowly than that of all the particles because non-fluorescent particles become unbound at the front of the perturbation making it spread out faster. We must remember that in this example the dynamics is effectively diffusive when observed over a long enough time. A transient non-diffusive behavior can in fact in be observed in all three plots in Fig. 2. As explained in supplementary text S3 in this case $\langle r^2 \rangle$ eventually becomes linear with t with a slope that is $2dD_{coll}$ if we consider all the particles and $2dD_{sm}$ if we only consider the fluorescent ones. Given the parameters of the simulation, it is $D_{coll} = 10\mu m^2/s$ and $D_{sm} = 0.7\mu m^2/s$. From the slopes of the curves in Figs. 2A and 2B we obtain diffusion coefficients $\approx 10\mu m^2/s$ and $\approx 1\mu m^2/s$, respectively. In text S3 we explain that $2dD_{sm}$ is also the constant of proportionality between the averaged mean square displacements of the added particles and time once the diffusive behavior sets in. This averaged MSD is shown in Fig. 1C. From its slope we obtain a diffusion coefficient $\approx 1.06\mu m^2/s$ which agrees with the one derived from Fig. 2B and is of the order of magnitude of the expected value, $D_{sm} = 0.7\mu m^2/s$.

In the examples discussed in the previous paragraph there is a site-mediated interaction between the particles and this explains why $D_{sm} < D_{coll}$. How large this difference is depends on the timescales and concentrations involved. There are two limits in which the site-mediated coupling does not affect differently the single particle and collective diffusion coefficients. If there are very few particles then most binding sites will be empty at any given time and the individual particles will not compete with one another for the sites. Most of the particles, on the other hand, will be bound to sites diffusing with diffusion coefficient, D_S (will stay immobile in the example). In this limit, $D_{sm} \approx D_{coll} \approx D_S$. The other limit holds when there are so many particles that almost all sites are occupied (bound). The effective diffusion coefficient is then determined by the very large number of particles that remain free. In this limit it is

$D_{sm} \approx D_{coll} \approx D_f$. Outside these limits the difference between D_{sm} and D_{coll} can be observable. The existence of one coefficient ruling the diffusion of individual particles and another one ruling the decay of concentration gradients also occurs in the context of non-ideal solutions [13] particularly those involving polymers [1, 14]. Also in this case it is the interaction between the diffusing particles that occurs when solutions are too concentrated or when the molecules are too large that causes concentration gradients to relax with one diffusion coefficient ("mutual" or "cooperative" diffusion) that differs form the one that characterizes the mean square displacement of individual particles ("self-diffusion") [13, 14]. It is important to point out that the existence of binding sites provides an interaction mechanism even for dilute solutions of very small molecules. We must also remember that the normal diffusive behavior only holds in the "long time limit", *i.e.*, if the mean-square displacement is computed over very long times or if the decay of a concentration perturbation is observed when it is reaching the new equilibrium condition.

IV. EFFECTIVE DIFFUSION, FRAP AND FCS

The previous discussion shows that indistinguishability and nonlinearity are related. Namely, by distinguishing the particles one can "break" nonlinearity and diffusion is then governed by the effective coefficient, D_{sm} that is obtained within the framework of linear equations [15]. Particles can be individualized if one could "tag" and follow them. In fact tagging is what underlies the applicability of optical techniques that use fluorescently labeled molecules. Tagging, however, not always induces distinguishability (*i.e.* if all molecules of interest are tagged). Thus, the interpretation of the transport rates that can be inferred from different optical techniques requires great care. In this paper we are interested in two techniques, Fluorescence Recovery After Photobleaching (**FRAP**) and Fluorescence Correlation Spectroscopy (**FCS**). The application of these techniques to study the dynamics of Bicoid involves working with transgenic embryos that express Bcd with an Enhanced-Green-Fluorescence-Protein (EGFP) tail [16]. Even if all Bcd had an EGFP tag, the fact that it takes a while for GFP to become mature and, thus, fluorescent, both fluorescent and non-fluorescent versions of Bcd-EGFP coexist in the embryos [17]. In Eqs. (6) of the accompanying paper we distinguish the fluorescent and non-fluorescent versions of the species involved by means of the superscripts t and u, respectively. In **FRAP** a region of interest is observed in which it is assumed that the various species that determine the dynamics of Bcd-EGFP are in equilibrium. At the beginning of the experiment, the region of interest is illuminated with an intense laser so as to bleach the fluorescence inside it. The fluorescence is subsequently recovered due to the transport of fluorescently labeled molecules into the region of interest. Fitting the fluorescence recovery curve one can obtain an estimate of the rate at which the fluorescent molecules are transported into the region. This entails having a model of the dynamics that underlies this recovery. If the bleaching occurs during a much shorter time than the one it takes for the fluorescent to recover, there is a simple analytic relationship between the recovery time and the transport rate [18–20]. If this is not the case, the interpretation of the observations requires a more sophisticated model [21]. In any case, if the particles diffuse and react with binding sites as in the model described by Eqs. (7), the recovery eventually occurs at a rate that is determined by D_{sm} [13, 22]. As explained in [12] and in supplementary text S2, the equations that rule the fluorescence recovery are linear. In such a case the effective coefficient is given by $D_f(t_f/t) + D_S(t_f/t) = D_{sm}$. The fact that D_{sm} is recovered in **FRAP** is illustrated in Video S3 and Fig. 3 of the accompanying manuscript. In **FCS** the time course of the fluorescence coming from a small observation volume is monitored. The dynamics of the fluorescence fluctuations around the mean is assessed by computing their auto-correlation function. In a system in which fluorescent particles diffuse and interact with binding sites without altering their emission, fluorescence fluctuations in an observation volume are mainly due to changes in the number of fluorescent particles inside it. Thus, information on the transport rate of the fluorescent particles can be inferred from the autocorrelation function. Unless the observation volume is very small, in the case in which particles react and diffuse **FCS** provides information on D_{coll} if all particles are fluorescent (and there is no distinguishability). If both fluorescent and non-fluorescent particles coexist, **FCS** gives information on both D_{coll} and D_{sm} [23]. For a more detailed explanation on **FCS**, see supplementary text S2.

[1] Höfling F, Franosch T (2013) Anomalous transport in the crowded world of biological cells. Reports on Progress in Physics 76: 046602.
[2] Einstein A (1956) Investigations on the Theory of the Brownian Movement. Courier Dover Publications.
[3] Einstein A (1989) The Collected Papers of Albert Einstein, Volume 2. Translated by Anna Beck, consultant Peter Havas. Princeton: Princeton University Press.
[4] Sokolov IM, Klafter J (2005) From diffusion to anomalous diffusion: A century after Einstein's Brownian motion. Chaos: An Interdisciplinary Journal of Nonlinear Science 15: 026103.
[5] Gardiner CW (1997) Handbook of Stochastic Methods for Physics, Chemistry and the Natural Sciences. Berlin, Heidelberg, New York: Springer-Verlag.
[6] Mendez V, Fedotov S, Horsthemke W (2010) Reaction-

Transport Systems. Mesoscopic Foundations, Fronts, and Spatial Instabilities. Berlin, Heidelberg, New York: Springer-Verlag.

[7] Banks DS, Fradin C (2005) Anomalous diffusion of proteins due to molecular crowding. Biophysical Journal 89: 2960 - 2971.

[8] Nagle J (1992) Long tail kinetics in biophysics? Biophysical Journal 63: 366 - 370.

[9] Saxton M (1994) Anomalous diffusion due to obstacles: a Monte Carlo study. Biophysical Journal 66: 394 - 401.

[10] Saxton M (1996) Anomalous diffusion due to binding: a Monte Carlo study. Biophysical Journal 70: 1250 - 1262.

[11] van Kampen N (2007) Stochastic processes in Physics and Chemistry. North-holland.

[12] Pando B, Dawson SP, Mak DOD, Pearson JE (2006) Messages diffuse faster than messengers. Proc Natl Acad Sci (USA) 103: 5338-5342.

[13] Petrov E, Schwille P (2008) State of the art and novel trends in fluorescence correlation spectroscopy. In: Resch-Genger U, editor, Standardization and Quality Assurance in Fluorescence Measurements II, Springer Berlin Heidelberg, volume 6 of *Springer Series on Fluorescence*. pp. 145-197. doi:10.1007/4243_2008_032.

[14] Zettl U, Hoffmann ST, Koberling F, Krausch G, Enderlein J, et al. (2009) Self-diffusion and cooperative diffusion in semidilute polymer solutions as measured by fluorescence correlation spectroscopy. Macromolecules 42: 9537-9547.

[15] Ipiña EP, Dawson SP (2013) Breaking nonlinearity with partial bleaching simplifies the analysis of biomolecule transport observations in intact cells. Publicaciones Matemáticas del Uruguay 14: 234-243.

[16] Gregor T, Wieschaus EF, McGregor AP, Bialek W, Tank DW (2007) Stability and nuclear dynamics of the bicoid morphogen gradient. Cell 130: 141 - 152.

[17] Little SC, Tkaik G, Kneeland TB, Wieschaus EF, Gregor T (2011) The formation of the bicoid morphogen gradient requires protein movement from anteriorly localized mrna. PLoS Biol 9: e1000596.

[18] Brown EB, Wu ES, Zipfel W, Webb WW (1999) Measurement of molecular diffusion in solution by multiphoton fluorescence photobleaching recovery. Biophysical Journal 77: 2837 - 2849.

[19] Soumpasis D (1983) Theoretical analysis of fluorescence photobleaching recovery experiments. Biophys J 41: 95–97.

[20] Stasevich TJ, Mueller F, Michelman-Ribeiro A, Rosales T, Knutson JR, et al. (2010) Cross-validating {FRAP} and {FCS} to quantify the impact of photobleaching on in vivo binding estimates. Biophysical Journal 99: 3093 - 3101.

[21] Braga J, Desterro JM, Carmo-Fonseca M (2004) Intracellular macromolecular mobility measured by fluorescence recovery after photobleaching with confocal laser scanning microscopes. Molecular Biology of the Cell 15: 4749-4760.

[22] Sprague BL, Pego RL, Stavreva DA, McNally JG (2004) Analysis of binding reactions by fluorescence recovery after photobleaching. Biophysical Journal 86: 3473 - 3495.

[23] Sigaut L, Ponce ML, Colman-Lerner A, Dawson SP (2010) Optical techniques provide information on various effective diffusion coefficients in the presence of traps. Phys Rev E 82: 051912.
