# Supplementary material for: Messages Do Diffuse Faster than Messengers: Reconciling Disparate Estimates of the Morphogen Bicoid Diffusion Coefficient
Source: PLoS Comput Biol. 2014 Jun 5;10(6):e1003629. doi: 10.1371/journal.pcbi.1003629 (PMC4046929; doi:10.1371/journal.pcbi.1003629)
Supplement: Text S2 — Detailed description of the mapping between model and experiments. In this text we discuss the main assumptions that underlie the application of our simple biophysical model to interpret the results obtained from FCS and FRAP experiments performed in Drosophila embryos that express Bcd-EGFP. More specifically we compare different possible mappings, discuss the compatibility of the models assumptions with the observations, their limitations and possible extensions. (PDF) [file pcbi.1003629.s002.pdf]

Text S2: Detailed description of the mapping between model and experiments.

I. UNDERLYING BIOPHYSICAL MODEL

We consider the simplest biophysical model model that incorporates the presence of molecules, S, that interact with the Bicoid protein according to the scheme:

$$Bcd_f + S \underset{k_{off}}{\overset{k_{on}}{\rightleftharpoons}} Bcd_b \tag{1}$$

with dissociation constant, $K_D = k_{off}/k_{on}$. In Eq. (1) Bcd_f stands for Bcd in its free form and Bcd_b for the $bcd - S$ complex. We assume that the S molecules are more massive than the free Bcd molecules in such a way that both S and Bcd_b have the same diffusion coefficient, D_S, which is in turn smaller than the coefficient of free Bcd molecules, D_f. We assume that all three species are, on average, uniformly distributed within the volume of interest for FCS and FRAP experiments. This approximation is good in the present case. Namely, the typical lengthscale of the Bcd gradient is of the order of 0.4 times the length of the embryo (*i.e.*, $\sim 150\mu m$), the size of the region from which fluorescence is collected in FCS and FRAP experiments is $\sim 0.4\mu m$ and $\sim 0.95\mu m$, respectively [1] and the size of the region from which Bcd molecules could come into the observation volume during the time course of the experiments is smaller than $9\mu m$. Therefore, it is safe to assume a spatially uniform distribution of the mean concentration values of all species. We call Bcd_T and S_T, the total mean concentrations (*i.e.*, both in their free and bound forms) of Bcd and S, respectively. We assume that, during the time course of the experiments, Bcd and S are in equilibrium, so that their mean concentrations are given by the equilibrium concentrations, Bcd_{feq}, Bcd_{beq} and S_{eq} which satisfy

$$Bcd_{feq} + Bcd_{beq} = Bcd_T, \tag{2}$$
$$S_{eq} + Bcd_{beq} = S_T, \tag{3}$$
$$Bcd_{feq} S_{eq} = K_D Bcd_{beq}. \tag{4}$$

Using all these conditions we obtain:

$$Bcd_{feq} = \frac{K_D Bcd_T}{K_D + S_{eq}}, \tag{5}$$
$$Bcd_{beq} = \frac{S_{eq} Bcd_T}{K_D + S_{eq}}, \tag{6}$$
$$\frac{Bcd_T/S_{eq}}{K_D/S_{eq} + 1} + 1 = \frac{S_T}{S_{eq}}. \tag{7}$$

Eq. (7) can be used to write Bcd_{feq}, Bcd_{beq} and S_{eq} as functions of S_T and Bcd_T. Following [2], we will refer to S as *traps* and to Bcd molecules as *particles*.

In order to assess the rate at which Bcd diffuses *in vivo*, FCS and FRAP experiments are done using embryos that only express Bcd- GFP. However, it takes some time for GFP to become mature and, thus, fluorescent. Therefore, we will consider that both fluorescent and non-fluorescent Bcd molecules coexist in the system. We will use the superscript t to indicate that the molecule is fluorescent (*e.g.* is "tagged") and the superscript u to indicate that is not. Therefore, the problem that we are dealing with involves the following 5 species: Bcd_f^t, Bcd_f^u, Bcd_b^t, Bcd_b^u and S, where the corresponding equilibrium concentrations satisfy: $Bcd_{feq}^t + Bcd_{beq}^t = Bcd_T^t$, $Bcd_{feq}^t + Bcd_{beq}^t + Bcd_{feq}^u + Bcd_{beq}^u = Bcd_T$ and $S_{eq} + Bcd_{beq}^t + Bcd_{beq}^u = S_T$. We will also analyze the limiting case in which all Bcd is fluorescent.

II. EFFECTIVE DIFFUSION COEFFICIENTS

The transport of particles in systems like the one we have just introduced can be described in terms of "effective" diffusion coefficients which take into account the effect of free diffusion and of reactions. Following [2] we define two such coefficients:

$$D_{sm} = \frac{D_f + \frac{S_{eq}}{K_D} D_S}{1 + S_{eq}/K_D}, \tag{8}$$

and

$$D_{coll} = \frac{D_f + \frac{S_{eq}^2}{K_D S_T} D_S}{1 + S_{eq}^2/(K_D S_T)}. \tag{9}$$

As shown in [2, 3], D_{sm} is the one that can be obtained from FRAP experiments if reactions occur on a fast timescale compared to diffusion. As shown in [4], D_{coll} can be derived from FCS experiments under the same condition. Furthermore, D_{sm}, can also be obtained with FCS experiments in certain cases [4].

III. FCS. THEORETICAL RESULTS

Given the biophysical model described in Sec. I it is possible to compute the quantities that are obtained in FCS and FRAP experiments. Moreover, under certain assumptions, analytic expressions can be derived for the relevant quantities. FCS monitors the fluorescence fluctuations in a small volume determined by the illuminated region which is commonly approximated by:

$$I(\mathbf{r}) = I(0) e^{\frac{-2r^2}{w_r^2}} e^{\frac{-2z^2}{w_z^2}}, \tag{10}$$

where $I(0)$ is the illumination intensity at $\mathbf{r} = 0$, (r, z) are cylindrical coordinates with z the spatial coordinate along the beam propagation direction and r a radial coordinate in the perpendicular plane and where w_z and w_r

are the sizes of the beam waist along z and r, respectively (in general, $w_z > w_r$). Fluctuations are characterized by the autocorrelation function (ACF):

$$G(\tau) = \frac{\langle \delta F(t) \delta F(t+\tau) \rangle}{< F(t) >^2} \tag{11}$$

where $< F(t) >$ is the average fluorescence in the volume and $\delta F(t)$ is the deviation with respect to this mean at each time, t.

In the case in which fluorescently labeled (Bcd) particles diffuse and interact with traps (S) according to the scheme (1), the fluorescence intensity is given by:

$$F(t) = \int QI(\mathbf{r})(Bcd_f^t(\mathbf{r},t) + Bcd_b^t(\mathbf{r},t))d^3r, \tag{12}$$

where $Bcd_f^t(\mathbf{r},t)$ ($Bcd_b^t(\mathbf{r},t)$) is the free (bound) fluorescently-labeled Bcd concentration at time, t, and spatial point, $\mathbf{r}$, and Q, takes into account the detection efficiency, the fluorescence quantum yield and the absorption cross-section at the wavelength of excitation of the fluorescence.

If all Bcd molecules are fluorescent (*i.e.*, $Bcd_f^t = Bcd_f$ and $Bcd_b^t = Bcd_b$ for all $\mathbf{r}$ and t),the ACF can be approximated by [4]:

$$\begin{aligned} G(\tau) &= \frac{Go_{ef}}{\left(1+\frac{\tau}{\tau_{coll}}\right)\sqrt{1+\frac{\tau}{w^2\tau_{coll}}}} \\ &+ \frac{Go_S}{\left(1+\frac{\tau}{\tau_S}\right)\sqrt{1+\frac{\tau}{w^2\tau_S}}}, \end{aligned} \tag{13}$$

where $\tau_S = w_r^2/(4D_S)$, $\tau_{coll} = w_r^2/(4D_{coll})$, $w = w_z/w_r$ and:

$$Go_S = \frac{(Bcd_{beq}^t)^2}{V_{ef}(Bcd_T^t)^2 S_T}, \tag{14}$$

$$\begin{aligned} Go_{ef} &= \frac{1}{V_{ef}Bcd_T} - \frac{(Bcd_{beq}^t)^2}{V_{ef}Bcd_T^2 S_T} \\ &= \frac{Bcd_{feq}^t}{V_{ef}(Bcd_T^t)^2}\left(1+\frac{S_{eq}^2}{K_D S_T}\right), \end{aligned} \tag{15}$$

with $V_{ef} = \pi w_r^2 w_z$ the effective volume.

If fluorescent and non-fluorescent (Bcd) particles coexist, with free and bound concentrations Bcd_f^t, Bcd_f^u, Bcd_b^t and Bcd_b^u, respectively (and with $Bcd_f^t + Bcd_f^u = Bcd_f$ and $Bcd_b^t + Bcd_b^u = Bcd_b$ for all $\mathbf{r}$ and t and mean or equilibrium values that satisfy: $Bcd_{feq}^t + Bcd_{beq}^t = Bcd_T^t$ and $Bcd_{feq}^u + Bcd_{beq}^u = Bcd_T^u$), the ACF can be approximated by [4]:

$$\begin{aligned} G(\tau) &= \frac{Go_{coll}}{\left(1+\frac{\tau}{\tau_{coll}}\right)\sqrt{1+\frac{\tau}{w^2\tau_{coll}}}} \\ &+ \frac{Go_{sm}}{\left(1+\frac{\tau}{\tau_{sm}}\right)\sqrt{1+\frac{\tau}{w^2\tau_{sm}}}} \\ &+ \frac{Go_S}{\left(1+\frac{\tau}{\tau_S}\right)\sqrt{1+\frac{\tau}{w^2\tau_S}}}, \end{aligned} \tag{16}$$

where

$$Go_{sm} = \frac{1-f}{V_{ef}Bcd_T^t}, \qquad Go_{coll} = fGo_{ef}, \tag{17}$$

Go_{ef}, Go_S, τ_S and τ_u are defined as before, $\tau_t = w_r^2/(4D_t)$ and $f \equiv Bcd_T^t/Bcd_T$ is the total fraction of labeled particles (so that $Bcd_T^u = (1-f)Bcd_T$).

The analytic approximations (13)–(16) hold as long as reactions are fast compared to the diffusion time-scale over the observation volume and if initial fluctuations in the number of molecules of each species involved can be assumed to obey Poisson statistics [5]. This last assumption does not hold for S and Bcd_b in the case of immobile traps given that $Bcd_b + S = S_T$ is fixed and, therefore, S and Bcd_b are anticorrelated. A binomial [6] or multinomial (because $S_T = Bcd_b^t + Bcd_b^u + S$) distribution should be used depending on whether all Bcd_b is fluorescent or not. The timescales remain the same as in the case of Poisson statistics but the various components have different weights. Taking into account the binomial or multinomial distributions accordingly, the weights read [7]:

$$Go_S = 0, \tag{18}$$

$$\begin{aligned} Go_{ef} &= \frac{1}{V_{ef}(Bcd_T)^2}\left(Bcd_{feq} + \frac{S_{eq}Bcd_{beq}}{S_T}\right) \\ &= \frac{Bcd_{feq}^t}{V_{ef}(Bcd_T^t)^2}\left(1+\frac{S_{eq}^2}{K_D S_T}\right), \end{aligned} \tag{19}$$

if all Bcd is fluorescent and:

$$Go_S = 0, \tag{20}$$

$$Go_{sm} = \frac{1-f}{V_{ef}(Bcd_T^t)}, \tag{21}$$

$$Go_{coll} = \frac{fBcd_{feq}^t}{V_{ef}(Bcd_T^t)^2}\left(1+\frac{S_{eq}^2}{K_D S_T}\right). \tag{22}$$

if fluorescent and non-fluorescent Bcd molecules coexist in the system. We then observe that using a binomial or multinomial statistics for Bcd_b^t we recover the same weights as in the case of Poisson statistics with the difference that $Go_S = 0$. In particular, inserting Eqs. (20)–(22) into Eq. (16) we obtain that, if traps are immobile and there are fluorescent and non-fluorescent Bcd

molecules in the system, the ACF can be approximated by:

$$G(\tau) = \frac{Go_{coll}}{\left(1+\frac{\tau}{\tau_{coll}}\right)\sqrt{1+\frac{\tau}{w^2\tau_{coll}}}} + \frac{Go_{sm}}{\left(1+\frac{\tau}{\tau_{sm}}\right)\sqrt{1+\frac{\tau}{w^2\tau_{sm}}}}. \quad (23)$$

In the case in which all variables satisfy Poisson statistics the sum of all the weights is equal to the inverse number of fluorescent particles in the observation volume. From Eqs.(14)–(15) and (17) we get:

$$Go_{ef} + Go_S = \frac{1}{V_{ef} Bcd_T},$$
$$Go_{coll} + Go_{sm} + Go_S = \frac{1}{f V_{ef} Bcd_T} = \frac{1}{V_{ef} Bcd_T^t} \quad (24)$$

This does not hold if Bcd_b^t does not obey Poisson statistics because in such case the variance and the mean of the number of bound fluorescent molecules are not equal [7]. Using Eqs. (18)–(22) we get:

$$Go_S + Go_{ef} = Go_{coll} + Go_{sm} + Go_S = \frac{1}{V_{ef}(Bcd_T^t)^2} \times \left(Bcd_{feq}^t + Bcd_{beq}^t\left(1 - \frac{Bcd_{beq}^t}{S_T}\right)\right). \quad (25)$$

The equation $Go_S = 0$ is exact if the traps are immobile. In most cases this is an approximation. In any case, this shows that there are uncertainties associated to the weights. For this reason, we derive most of our results only from the correlation times and not the weights determined from fits to the experimental ACF's.

Summarizing, the approximated ACF's have two components in the following cases:

- Case I: All Bcd particles are fluorescent and the only trap considered is mobile (Eq. (13)) ,
- Case II: There are fluorescent and non-fluorescent Bcd particles and the only trap considered is immobile (Eq. (23)) ,

while we have found an ACF with three components in the following case:

- Case III: There are fluorescent and non-fluorescent Bcd particles and the only trap considered is mobile (Eq. (16)) .

We only consider cases with two or three components because the fits presented in [1] were not good for a one component ACF. Case I, on the other hand, corresponds to a situation that is not expected to occur. Namely, the studies of [8] show that there is always a fraction of immature Bcd-EGFP during the stages at which FCS experiments are performed in *Drosophila* embryos. Furthermore, assuming Case I and working as we explain later we obtained estimates of the diffusion coefficients and concentrations that did not seem to be realistic. For this reason, we will only present the results of interpreting the published data in terms of Cases II and III.

The list of possible cases is exhaustive for the simple biophysical model that we are considering here. It is very likely that this model be an oversimplification of what actually happens. In particular, it is probable that there is more than one type of "trap". From the analysis we perform here we expect to extract information on typical parameter values for the simplest model compatible with the observations that can yet be used to understand the dynamics of the Bcd gradient formation.

IV. FRAP. THEORETICAL RESULTS

We show here that the equations that describe the spatio-temporal distribution of the various concentrations of interest in the case of FRAP experiments give the same answers whether, before photobleaching, there are fluorescent and non-fluorescent Bcd molecules in the same system or if all of them are fluorescent. The analysis of FRAP experiments does not take concentration fluctuations into account but rather works with mean values. We must then recall that at $t = 0$ (ideally) the sum of mean concentration values $Bcd_f^u + Bcd_f^t = Bcd_{feq}$, $Bcd_b^u + Bcd_b^t = Bcd_{beq}$ and $S = S_{eq}$ everywhere in space. Thus, $\partial(Bcd_f^u + Bcd_f^t)/\partial t|_{t=0} = \partial(Bcd_b^u + Bcd_b^t)/\partial t|_{t=0} = \partial S/\partial t|_{t=0} = 0$ so that $S = S_{eq}$, $Bcd_f^u + Bcd_f^t = Bcd_{feq}$, and $Bcd_b^u + Bcd_b^t = Bcd_{beq}$ for all time everywhere in space. Therefore, the 5 nonlinear coupled equations describing the evolution of Bcd_f^u, Bcd_f^t, Bcd_b^u, Bcd_b^t and S reduce analytically, as in [2], to the following linear equations:

$$\frac{\partial Bcd_f^t}{\partial t} = D_f \nabla^2 Bcd_f^t - k_{on} Bcd_f^t S_{eq} + k_{off} Bcd_b^t \quad (26)$$
$$\frac{\partial Bcd_b^t}{\partial t} = D_S \nabla^2 Bcd_b^t + k_{on} Bcd_f^t S_{eq} - k_{off} Bcd_b^t \quad (27)$$
$$\frac{\partial Bcd_f^u}{\partial t} = D_f \nabla^2 Bcd_f^u - k_{on} Bcd_f^u S_{eq} + k_{off} Bcd_b^u \quad (28)$$
$$\frac{\partial Bcd_b^u}{\partial t} = D_S \nabla^2 Bcd_b^u + k_{on} Bcd_f^u S_{eq} - k_{off} Bcd_b^u, \quad (29)$$

while $S = S_{eq}$ everywhere in space for all times. The only difference between having a mixture of fluorescent and nonfluorescent Bcd particles and having all of them fluorescent before inducing the photobleaching only changes the initial condition, but the eigenvalues describing the dynamics remain the same. This means that the timescale over which the fluorescence recovers after the bleaching pulse also remains the same.

V. DESCRIPTION OF PUBLISHED EXPERIMENTAL RESULTS

We here summarize the experimental results that we will interpret within the framework of the simple biophysical model introduced in Sec. I. The aim is to estimate the various parameters of the model, including diffusion coefficients, reaction rates and concentrations. In particular, we will look at some results of Abu-Arish *et al* [1],Porcher *et al* [9] and of Gregor *et al* [10].

In [1, 9] the mobility of two species is probed in embryos of *Drosophila melanogaster*: Bcd-EGFP and NLS-EGFP. As mentioned by the authors, NLS-EGFP forms a gradient that is similar to that of Bcd, but at the same time "it should be a mostly freely diffusing protein even if some transient interactions with nuclear transport factors are expected". Therefore, we expect to draw information on the free diffusion coefficient of Bcd-EGFP from the FCS experiments done with NLS-EGFP. The only difference between the NLS-EGFP diffusion coefficient and the free diffusion coefficient of Bcd that we consider is the one due to the different masses of both molecules. Namely, we use the Stokes-Einstein relation to obtain:

$$\frac{D_{Bcd-egfp}}{D_{NLS-egfp}} = \left(\frac{MW_{NLS-egfp}}{MW_{Bcd-egfp}}\right)^{1/3}, \tag{30}$$

where MW stands for the molecular weights of the corresponding molecules ($MW_{Bcd-egfp} = 80kDa$ and $MW_{NLS-egfp} = 30kDa$). We further assume that the labeling with EGFP does not alter the free diffusion coefficient of Bcd. Thus, we make the identification:

$$D_f = D_{Bcd-egfp} \tag{31}$$

between one of the model parameters and one that can be obtained from the experiments.

The FCS experiments that we are looking at were performed in anterior nuclei during cycles 13 and 14 [9] and in the anterior cortical cytoplasm during interphase at stage $12--14$ [1] from which the ACF was computed. In all cases, the experimental ACF was fitted by a function of the general form:

$$G(\tau) = \frac{1}{N}\left(1+\frac{B}{1-B}e^{-\tau/\tau_B}\right) \times \sum_{j=1}^{n} \frac{F^{(j)}}{\left(1+\left(\frac{\tau}{\tau^{(j)}}\right)^{\alpha_j}\right)\sqrt{1+\left(\frac{\tau}{w^2\tau^{(j)}}\right)^{\alpha_j}}}, \tag{32}$$

where B is the fraction of fluorophore molecules in the dark state, τ_B is the relaxation time of the fluorophore blinking process, n is the number of components being considered and the exponent α_j characterizes the transport process of the j-th component ($\alpha_j = 1$, $\alpha_j < 1$ and $\alpha_j > 1$ for normal, sub- and super- diffusion, respectively). For the Bcd-EGFP experiments performed in the cytoplasm, the authors tried various fits. In some of them, they assumed fixed values of B and τ_B *a priori* ($B = 0.2$, $\tau_B = 0.22ms$) and in others they included them among the fitting parameters. From now on we will restrict ourselves to analyzing the results they obtained setting $B = 0.2$, $\tau_B = 0.22ms$. Then, they fitted their data with either $n = 1, 2$ or 3 components assuming that $\alpha_j = 1$ for all the components or including the α_j's among the fitting parameters. Keeping the values of the α_j's free did not improve the quality of the fits (as measured by the normalized chi-square function, Ξ^2/ν) [1]). We will only consider the results obtained with $\alpha_j = 1$ for all j's. Based on its quality, it is clear that the fitting with $n = 1$ and $\alpha_1 = 1$ is not very good and we will not consider this case either. For the experiments performed in nuclei (both for Bcd and NLS-EGFP) [9] and for the NLS-EGFP experiments performed in the cytoplasm [1], the authors only present results with $n = 2$ and $\alpha_j = 1$ for all j's. From the various fits, the authors give tables with the values of $\tau^{(j)}$ (mean residence time of the j-th component in the detection volume) and $F^{(j)}$ (fraction of molecules of the j-th component in the detection volume) that they obtain for the various cases considered [1, 9]. $\tau^{(j)}$ can be transformed into a (usually effective) diffusion coefficient by: $D^{(j)} = w_r^2/(4\tau^{(j)})$ with w_r as in Eq. (10). From a previous calibration, the values $w_r = (0.40 \pm 0.03)\mu m$ and $w_z = (2 \pm 0.5)\mu m$ are determined from which the ratio $w = w_r/w_z = 0.2$ is obtained [1]. The values of the various τ_j that were obtained in all the cases that we are analyzing here are much larger than τ_B. Therefore, we can assume that for τ of the order or larger than the smallest τ_j the term $B/(1 - B\exp(-\tau/\tau_B)) \ll 1$. So, we can replace the factor $1 + B/(1 - B\exp(-\tau/\tau_B))$ by one in Eq. (32) for the values of τ for which the ACF becomes "interesting". Regarding N, the average number of molecules in the detection volume, (which is not listed in the Table included in the supplementary material of [1]), the authors infer its value from measurements done inside the nuclei at the beginning of nuclear cycle 14. The concentration of fluorescent particles inside nuclei and in the cytoplasm during interphase is different (as may be observed in [10]). Furthermore, N varies between 20 and 150 and the measurements are very sensitive to errors in the calibration of the detection volume which is determined only with a $\sim 50\%$ precision [1]). These comments show that only a rough estimate of N could be obtained from the experiments.

We also consider some results obtained in embryos of *Drosophila melanogaster* using the FRAP technique [1, 10]. The FRAP experiments that we will analyze were performed in the cortical cytoplasm during the mitosis following nuclear cycles 12 or 13. In both these works, the authors fit the recovery curve assuming that the photobleaching flash is instantaneous. No fitting functions are presented in [1, 10]. However, based on the references cited in those papers we suppose that the fitting

of [10] is of the form [11]:

$$F(t) = F_0 \sum_{\ell \leq 0} \frac{m^{3/2}(-\beta)^\ell}{\ell!} \frac{1}{m + b\ell + (b\ell m t/\tau_D)} \times \frac{1}{\sqrt{m + b\ell + (b\ell m t/(R\tau_D))}} \quad (33)$$

where $\tau_D = w_r^2/(8D_{FRAP})$ with D_{FRAP} the diffusion coefficient that can be obtained with this technique, $R = w_z^2/w_r^2$, w_r and w_z as in (10), m the number of photons required to generate a fluorescence photon, b the number of photons absorbed in a bleaching event, β the bleach depth parameter that depends on the bleaching action cross section, the average of the peak intensity at the center of the focal spot and the bleaching pulse duration. In [1] we suppose that the fitting is of the form [3, 12]:

$$F_R(t) \equiv \frac{F(t) - F(0)}{F(\infty) - F(0)} = \exp(-\tau_D/2t)\left(I_o\left(\frac{\tau_D}{2t}\right) + I_1\left(\frac{\tau_D}{2t}\right)\right) \quad (34)$$

with I_0 and I_1 modified Bessel functions and $\tau_D = w^2/D_{FRAP}$. Eq. (33) holds for diffusion in three space dimensions and Eq. (34) for diffusion in two dimensions. Even if an area of radius $0.95\mu m$ is scanned during the photobleaching process in the experiments of [1] it is actually a volume that is photobleached the thickness of which is of the order of $1\mu m$ for a confocal microscope. Given that Bcd diffuses in three dimensions, using a fitting function that holds for two dimensions instead of three can lead to an overestimate of the diffusion coefficient. On the other hand, the delay in the bleaching and in the acquisition that scanning an area with a confocal microscope introduces can lead to an underestimation of the coefficient as discussed in [1, 13]. These two artifacts lead to opposite effects. For this reason we think that the estimate, D_{FRAP}, derived in [1] is not off by over an order of magnitude with respect to its actual value.

Tables I, II and III list the main experimentally determined parameters that we will consider.

We also use some results of [10]. In particular, from Figs. 3 and 4 of this paper we extract the following approximate relations between some concentrations of interest in different regions and/or situations:

$$\frac{[\mathrm{Bcd-EGFP}]^{nuc}}{[\mathrm{Bcd-EGFP}]^{intercyt}} \sim 2.3 - 4.5, \qquad \frac{[\mathrm{Bcd-EGFP}]^{mito}}{[\mathrm{Bcd-EGFP}]^{intercyt}} \sim 1.2, \quad (35)$$

where $[\mathrm{Bcd-EGFP}]^{nuc}$ and $[\mathrm{Bcd-EGFP}]^{intercyt}$ correspond to the total concentration of fluorescent Bcd in the nucleus and in the cytoplasm during interphase, respectively, and $[\mathrm{Bcd-EGFP}]^{mito}$ to the same concentration during mitosis (in the cytoplasm). Using the range of possible values of $[\mathrm{Bcd-EGFP}]^{nuc}$ estimated in [1] and considering $[\mathrm{Bcd-EGFP}]^{nuc}/[\mathrm{Bcd-EGFP}]^{intercyt} \approx$ 2.4 we obtain the ranges of concentrations values listed in Table IV. The ratio between $[\mathrm{Bcd-EGFP}]^{nuc}$ and $[\mathrm{Bcd-EGFP}]^{intercyt}$ varies within the range given in (35) as interphase proceeds (see *e.g.* Fig. 3 of [10]). The value 4 reported in Fig. 4 of [10] corresponds to a certain stage during this process. The ratios of concentrations and/or dissociation constant that we list in Table VI are independent of the value that we as-

TABLE I: Parameters obtained from FCS experiments performed in the cytoplasm during interphase by fitting the experimental ACF by an expression of the form (32) with $\alpha_j = 1$ and $n = 2$ or $n = 3$ [1].

**FCS (interphase, cytoplasm)**	
Bcd-EGFP	
Parameters obtained with an $n = 3$ fit	
Diffusion coefficient	Relative Weight
$D^{(1)} = (14 \pm 2)\ \mu m^2/s$	$F^{(1)} = (63 \pm 8)$ %
$D^{(2)} = (1.6 \pm 0.5)\ \mu m^2/s$	$F^{(2)} = (32 \pm 6)$ %
$D^{(3)} = (0.095 \pm 0.037)\ \mu m^2/s$	$F^{(3)} = (5 \pm 2)$ %
Parameters obtained with an $n = 2$ fit	
Diffusion coefficient	Relative Weight
$D^{(1)} = (8.9 \pm 0.4)\ \mu m^2/s$	$F^{(1)} = (82 \pm 1)$ %
$D^{(2)} = (0.38 \pm 0.03)\ \mu m^2/s$	$F^{(2)} = (18 \pm 1)$ %
NLS-EGFP	
Parameters obtained with an $n = 2$ fit	
Diffusion coefficient	Relative Weight
$D^{(1)} = (26.5 \pm 0.9)\ \mu m^2/s$	$F^{(1)} = (89 \pm 1)$ %
$D^{(2)} = (1.0 \pm 0.1)\ \mu m^2/s$	$F^{(2)} = (11 \pm 1)$ %

TABLE II: Parameters obtained from FRAP experiments performed in the cytoplasm during mitosis fitting the recovery curve with an expression along the lines of Eq. (33).

**FRAP (mitosis)**	
D_{FRAP}= [0.37 - 1] $\mu m^2/s$	[1]
D_{FRAP}= (0.30 ± 0.09) $\mu m^2/s$	[10]

TABLE III: Parameters obtained from FCS experiments performed in nuclei by fitting the experimental ACF by an expression of the form (32) with $\alpha_j = 1$ and $n = 2$ [9].

**FCS (nuclei)**	
Bcd-EGFP	
Parameters obtained with an $n = 2$ fit	
Diffusion coefficient	Relative Weight
$D^{(1)} = (7.7 \pm 0.3)\ \mu m^2/s$	$F^{(1)} = (57 \pm 1)$ %
$D^{(2)} = (0.22 \pm 0.01)\ \mu m^2/s$	$F^{(2)} = (43 \pm 1)$ %
NLS-EGFP	
Parameters obtained with an $n = 2$ fit	
Diffusion coefficient	Relative Weight
$D^{(1)} = (28 \pm 1\ )\ \mu m^2/s$	$F^{(1)} = (96 \pm 1\ )$ %
$D^{(2)} = (0.51 \pm 0.04\ )\ \mu m^2/s$	$F^{(2)} = (4 \pm 1)$ %

sume for $[\mathrm{Bcd-EGFP}]^{nuc}/[\mathrm{Bcd-EGFP}]^{intercyt}$. The absolute values that we list in Tables VIII–IX are not. Comparing the results that we obtain using different values of $[\mathrm{Bcd-EGFP}]^{nuc}/[\mathrm{Bcd-EGFP}]^{intercyt}$ within the range given in (35) we obtain the most consistent results for $[\mathrm{Bcd-EGFP}]^{nuc}/[\mathrm{Bcd-EGFP}]^{intercyt} = 2.4$. That is why we used that value in Table IV. Based on the range of concentrations, $[\mathrm{Bcd-EGFP}]^{intercyt}$, of Table IV we obtain the values of Tables VIII–IX. The absolute values of the concentration and dissociation constant obtained using another estimate for $r \equiv [\mathrm{Bcd-EGFP}]^{nuc}/[\mathrm{Bcd-EGFP}]^{intercyt}$ can be drawn directly from the ones listed in Tables VIII–IX by multiplying them by $2.4/r$.

A comparison of Eq. (32) when $1 + B/(1 - B\exp(-\tau/\tau_B)) \approx 1$ and $\alpha_j = 1$ for all j's and of Eqs. (13), (16) and (23) shows that they all have the same τ dependence. The main difference is that our calculations provide analytic expressions for the weights and residence times in terms of the biophysical parameters of the simple model that we assume underlies the observations. Therefore, given the weights and residence times determined experimentally in [1, 9], we expect to be able to derive values for at least some of the parameters of our simple biophysical model. It is clear that, depending on the situation that we consider (Cases I through III listed before) the mapping between the numbers obtained in [1, 9] and the biophysical parameters of the simple model is different. In all the cases, however, there is enough information to determine all the parameters of the biophysical model. We discuss the values that we obtain for each case in the following Sections and which of all the alternatives is the most plausible one.

VI. USING THE CORRELATION TIMES DERIVED FROM FCS EXPERIMENTS.

We use the diffusion coefficients obtained with the NLS-EGFP FCS experiments to estimate the free diffusion coefficient of Bcd-EGFP. These experiments were fitted in [1, 9] using a two component ACF. The second component, however, has a very small weight in both cases (see Tables I and III). Therefore, we assume that NLS-EGFP does not interact with binding sites and that its free diffusion coefficient is the one that can be extracted from the component with the largest weight.

TABLE IV: Range of total concentration values of fluorescent Bcd in the nucleus, in the cytoplasm during interphase and during mitosis, respectively, using estimates from [1] and [10] and setting $[\mathrm{Bcd-EGFP}]^{nuc}/[\mathrm{Bcd-EGFP}]^{intercyt} \approx 2.4$.

$[\mathrm{Bcd-EGFP}]^{nuc}$	[19 – 140] nM
$[\mathrm{Bcd-EGFP}]^{intercyt}$	[8 – 58] nM
$[\mathrm{Bcd-EGFP}]^{mito}$	[9.5 – 70] nM

TABLE V: Estimates of free and effective diffusion coefficients derived from FCS experiments performed in the cytoplasm and in nuclei using a two (Case II) or three (Case III) component ACF.

**Interphase, FCS location**			
	**Cytoplasm**		**Nucleus**
$[\mu m^2/s]$	**Case II**	**Case III**	**Case II**
D_f	19	19	20
D_S	0	0.095	0
D_{sm}	0.38	1.6	0.22
D_{coll}	8.9	14	7.7

This gives $D_{NLS-egfp} \approx (26.5 \pm 0.9)\mu m^2/s$ for the experiments performed in the cytoplasm during interphase (see Table I) and $D_{NLS-egfp} \approx (28\pm1)\mu m^2/s$ for the experiments performed in nuclei (see Table III). These two values are very similar. Inserting $MW_{Bcd-egfp} = 80kDa$, $MW_{NLS-egfp} = 30kDa$ and $D_{NLS-egfp}$= 26.5 $\mu m^2/s$ in Eq. (30) we obtain

$$D_f = D_{Bcd} \sim 19\mu m^2/s. \tag{36}$$

If we use $D_{NLS-egfp}$= 28 $\mu m^2/s$ instead we obtain $D_f \approx 20\mu m^2/s$.

For the case of Bcd-EGFP, the mapping between the parameters of our ACF's (Eqs. (23) or (16)) and those of Abu-Arish ([1] for experiments performed in the cytoplasm and [9] for experiments performed in nuclei) is done by associating the 2 or 3 components depending on the relative ordering of their diffusing times. Namely, for experiments performed in the cytoplasm, considering the results with the 3-component fit of [1] (see Table I) and assuming it corresponds to our Case III we obtain: $D_S = (0.095 \pm 0.037)\mu m^2/s$, $D_{sm} = (1.6 \pm 0.5)\mu m^2/s$, $D_{coll} = (14 \pm 2)\mu m^2/s$. Considering the results with the 2-component fit of [1] (also see Table I) and assuming it corresponds to our Case II we obtain: $D_{sm} = (0.38 \pm 0.03)\mu m^2/s$ and $D_{coll} = (8.9 \pm 0.4)\mu m^2/s$. The ranges of values of D_{coll} and D_{sm} that we obtain in both cases do not overlap. If we consider the full range defined by both fits we obtain $8.5\mu m^2/s \leq D_{coll} \leq 16\mu m^2/s$ and $0.3\mu m^2/s \leq D_{sm} \leq 2.1\mu m^2/s$. Finally, for experiments performed in nuclei, only the results of a 2 component fit are presented. In this case, mapping these results to the ACF of our Case II we obtain: $D_{sm} = (0.22 \pm 0.01)\mu m^2/s$, $D_{coll} = (7.7 \pm 0.3)\mu m^2/s$. The condition $D_S = 0$ is implicit in all the calculations involving Case II. The mean values corresponding to these results are listed in Table V.

Once we have D_f, D_S and D_{sm} we can use Eqs. (8)–(9) to determine the following ratios:

$$\begin{aligned} \alpha &\equiv \frac{S_{eq}}{K_D} = (D_f - D_{sm})/(D_{sm} - D_S); \quad , \\ \gamma &\equiv \frac{S_{eq}^2}{K_D S_T} = (D_f - D_{coll})/(D_{coll} - D_S) \quad , \\ \beta &\equiv \frac{S_{eq}}{S_T} = \frac{\gamma}{\alpha}, \end{aligned} \tag{37}$$

TABLE VI: Parameter estimates derived from the residence times of the ACF's obtained with FCS experiments performed in the cytoplasm or in nuclei during interphase. We will later identify these concentrations with the superscripts c, FCS or n, FCS to indicate that they correspond to cytoplasmic or nuclear estimates, respectively, during interphase at the location where FCS experiments were performed.

**Interphase, FCS location**			
	**Cytoplasm**		**Nucleus**
	**Case II**	**Case III**	**Case II**
S_{eq}/Bcd_T	0.023	0.03	0.018
Bcd_{beq}/Bcd_T	0.98	0.92	0.99
Bcd_{feq}/Bcd_T	0.02	0.08	0.01
S_T/Bcd_T	1.003	0.95	1.007
K_D/Bcd_T	$5\ 10^{-4}$	0.0026	0.0002

and combining these expressions with Eqs. (2)–(4) derive the ratio of the concentrations and of K_D with respect to any concentration, *e.g.*, Bcd_T:

$$\begin{aligned}\frac{Bcd_{feq}}{Bcd_T} &= (1+\alpha)^{-1},\\ \frac{Bcd_{beq}}{Bcd_T} &= \frac{\alpha}{(1+\alpha)},\\ \frac{S_{eq}}{Bcd_T} &= \frac{\gamma}{(1+\alpha)(1-\beta)},\\ \frac{S_T}{Bcd_T} &= \frac{\alpha}{(1+\alpha)(1-\beta)},\\ \frac{K_d}{Bcd_T} &= \frac{\beta}{(1+\alpha)(1-\beta)}. \end{aligned} \tag{38}$$

We list the mean values obtained using Eqs. (38) for experiments performed in the cytoplasm considering Case II and Case III and those for experiments performed in nuclei considering Case II in Table VI.

Notice that the values of Bcd_{feq}, Bcd_{beq} and Bcd_T in Table VI include both fluorescent and non-fluorescent Bcd molecules. We must finally mention that, since Bcd is non-uniformly distributed along the embryo axis, the estimates of Table VI only hold at the location where FCS experiments were performed. We will later identify these concentrations with the superscript c, FCS to highlight this feature and distinguish them from estimates that could hold at other locations.

VII. COMPARING THE RESULTS OF FCS EXPERIMENTS PERFORMED IN NUCLEI AND IN THE CYTOPLASM.

We can compare the concentrations that we obtain in the nucleus with those obtained in the cytoplasm during interphase in Case II. In particular, considering that K_D is the same in the cytoplasm and in the nucleus, we can compute $Bcd_T^{n,FCS}/Bcd_T^{c,FCS} = (Bcd_T^{n,FCS}/K_D)\ (K_D/Bcd_T^{c,FCS})$ using the results of Table VI. We obtain $Bcd_T^{n,FCS}/Bcd_T^{c,FCS} = 2.37$ which is within the range estimated from the figures of [10] given in Eqs. (35). This is the reason why we use $Bcd_T^{n,FCS}/Bcd_T^{c,FCS} = 2.4$ to generate the results displayed in Tables VIII–IX. Again using the results of Table VI and $Bcd_T^{n,FCS}/Bcd_T^{c,FCS} = 2.4$ we can compute $S_T^{n,FCS}/S_T^{c,FCS} = (S_T^{n,FCS}/Bcd_T^{n,FCS})$ $(Bcd_T^{n,FCS}/Bcd_T^{c,FCS})$ $(Bcd_T^{c,FCS}/S_T^{c,FCS})$. We obtain $S_T^{n,FCS}/S_T^{c,FCS} = 2.41$ which is almost the same as the value we obtained for $Bcd_T^{n,FCS}/Bcd_T^{c,FCS}$. As explained in the main body of the paper, this is an indication that the change in concentration can be due to a change in the available volume.

VIII. COMPARING THE RESULTS OF FCS AND FRAP EXPERIMENTS.

The values of Tables V-VI can be contrasted with the results obtained with FRAP experiments performed in the cytoplasm during mitosis. The effective diffusion coefficient determined in these FRAP experiments, D_{FRAP}, varied between $0.3\mu m^2/s$ [10] and 1 $\mu m^2/s$) [1]. The theory indicates that this measurement should correspond to D_{sm}, as given by Eq. (8) [2, 3]. Now, one of the effective diffusion coefficients extracted from FCS experiments is also D_{sm}. The numerical values obtained in the various cases probed, however, could differ since they depend on the concentration of the reactants and they could vary between interphase and mitosis and between nuclei and cytoplasm. In order to estimate how different the cytoplasmic concentrations during interphase or mitosis should be in order to obtain the values of D_{sm} determined with the FCS and FRAP experiments performed in the cytoplasm, we assume that the trap that is present in the cytoplasm during interphase and during mitosis is the same (the same K_D) and that the free diffusion coefficients (D_f and D_S) are also the same in both cases. The latter assumption is consistent with the similarity in the diffusion coefficients of NLS-EGFP determined in the cytoplasm and in nuclei. We assume, however, that all the concentrations could be different. In order to check the plausibility of describing the experimental results within the simple biophysical model introduced in Sec. I under this assumption, we estimate here how different the concentrations should be to accommodate the results of FCS and FRAP within our unified approach. To this end we compute α as in Eqs. (37) using the free diffusion coefficients, D_f and D_S of Table V and the value, D_{sm}, obtained in FRAP during mitosis (D_{sm}^{FRAP}). We have repeated the calculation for the two values of D_{sm}^{FRAP} reported in the literature: $0.3\mu m^2/s$ [10] and $1\mu m^2/s$ [1] and for the two values of D_S ($D_S = 0$ and $D_S = 0.095\mu m^2/s$) always keeping $D_f = 19\mu m^2/s$. In this way we obtain a set of possible values for $\alpha^{FRAP} = S_{eq}^{FRAP}/K_D$. Assuming that the only difference between these ratios and those obtained from FCS experiments in the cytoplasm

during interphase is the value of S_{eq} we can then obtain a set of values for the ratios $S_{eq}^{FRAP}/S_{eq}^{c,FCS}$ where we use the superscripts $FRAP$ and c, FCS to distinguish concentration estimates that correspond to the cytoplasm during mitosis and during interphase, respectively, at the location where FRAP and FCS experiments are performed. These two types of experiments are performed at approximately the same region along the axis of the embryo. Therefore, we think that the concentration changes we derive in this way are merely due to the transition from interphase to mitosis and not to a change of location along the axis. The ratios obtained cam be combined with the results of Table VI and with the estimates of (35) to obtain how much S_{eq} and S_T in the cytoplasm vary between interphase (as probed by FCS) and mitosis (as probed by FRAP). To this end we compute $(Bcd_T/S_{eq})^{FRAP} = (Bcd_T^{FRAP}/Bcd_T^{c,FCS})(Bcd_T^{c,FCS}/S_{eq}^{c,FCS})(S_{eq}^{c,FCS}/S_{eq}^{FRAP})$ taking the first ratio on the r.h.s. from Eq. (35) (assuming that $Bcd_T^{FRAP}/Bcd_T^{c,FCS} = [\mathrm{Bcd-EGFP}]^{mito}/[\mathrm{Bcd-EGFP}]^{intercyt} = 1.2$), the second from Table VI and the third from the ratios $S_{eq}^{FRAP}/S_{eq}^{c,FCS}$ derived as explained before. We then insert in Eq. (7) the obtained value of $(Bcd_T/S_{eq})^{FRAP}$ and the ratios, $\alpha^{FRAP} = S_{eq}^{FRAP}/K_D$ derived from D_{sm}^{FRAP}. Given that we have considered two possible values for D_S and two possible values for D_{sm}^{FRAP}, we obtain 4 possible values for $S_T^{FRAP}/S_T^{c,FCS}$:

$$\frac{S_T^{FRAP}}{S_T^{c,FCS}} = 1.21, \quad D_{sm}^{FRAP} = 0.3, \quad D_S = 0, \tag{39}$$

$$\frac{S_T^{FRAP}}{S_T^{c,FCS}} = 1.14, \quad D_{sm}^{FRAP} = 1, \quad D_S = 0, \tag{40}$$

$$\frac{S_T^{FRAP}}{S_T^{c,FCS}} = 1.49, \quad D_{sm}^{FRAP} = 0.3, \quad D_S = 0.095, \tag{41}$$

$$\frac{S_T^{FRAP}}{S_T^{c,FCS}} = 1.26, \quad D_{sm}^{FRAP} = 1, \quad D_S = 0.095, \tag{42}$$

where all the diffusion coefficients are given in $\mu m^2/s$. The ratios (39)–(42) have very reasonable values since they are similar to the ratio of [Bcd–EGFP] between mitosis and interphase (see Eqs. (35)). Given that these results have been obtained under the assumption that $(Bcd_T^{FRAP}/Bcd_T^{c,FCS}) = [\mathrm{Bcd-EGFP}]^{mito}/[\mathrm{Bcd-EGFP}]^{intercyt} = 1.2$ having approximately the same value for $(S_T^{FRAP}/S_T^{c,FCS})$ can be interpreted very simply as due to a change in the available volume between mitosis and interphase.

Finally, we can combine the values of $S_T^{FRAP}/S_T^{c,FCS}$ of Eqs. (39)–(42) with those of $S_T^{c,FCS}/Bcd_T^{c,FCS}$ of Tables VI to obtain $S_T^{FRAP}/Bcd_T^{c,FCS}$ for Cases II and III. We must remember that this result is derived under the assumption that $Bcd_T^{FRAP}/Bcd_T^{c,FCS} = 1.2$ and that $K_D/Bcd_T^{c,FCS}$ is the same during mitosis and interphase if we remain at the same region along the axis of the embryo. Knowing $S_T^{FRAP}/Bcd_T^{c,FCS}$, $Bcd_T^{FRAP}/Bcd_T^{c,FCS}$ and $K_D/Bcd_T^{c,FCS}$ (which we get from Table VI) we can compute the ratio of all the concentrations during mitosis at the location where FRAP and FCS experiments are performed with respect to $Bcd_T^{c,FCS}$. We list the results obtained for $D_{sm}^{FRAP} = 1\mu m^2/s$ in Table VII.

TABLE VII: Parameter estimates derived combining results from FRAP and FCS experiments and assuming $D_{sm}^{FRAP} = 1\mu m^2/s$

**Mitosis**		
	**Case II**	**Case III**
$S_{eq}^{FRAP}/Bcd_T^{c,FCS}$	0.009	0.05
$Bcd_{beq}^{FRAP}/Bcd_T^{c,FCS}$	1.14	1.14
$Bcd_{feq}^{FRAP}/Bcd_T^{c,FCS}$	0.06	0.06
$S_T^{FRAP}/Bcd_T^{c,FCS}$	1.15	1.19
$Bcd_T^{FRAP}/Bcd_T^{c,FCS}$	1.2	1.2

IX. ESTIMATING EFFECTIVE DIFFUSION COEFFICIENTS ALONG THE EMBRYO.

We know that Bcd_T changes along the axis of the embryo, so that the ratios of concentrations of Tables VI and VII and, consequently, the effective diffusion coefficients, D_{sm} and D_{coll} given by Eqs. (8)–(9), could vary along the axis too. Let us call x the spatial coordinate along the axis of the embryo and x_{FCS} the value of x where the FCS and the FRAP experiments were done and let us use the superscripts *intercyt* and *mito* to indicate that a quantity corresponds to the cytoplasm during interphase and mitosis, respectively, as done in Eqs. (35) and Table IV. Although there is not a single value of x_{FCS}, we can roughly say that $Bcd_T^{intercyt}(x_{FCS}) = Bcd_T^{c,FCS}$, $S_T^{intercyt}(x_{FCS}) = S_T^{c,FCS}$, $Bcd_T^{mito}(x_{FCS}) = Bcd_T^{FRAP}$ and $S_T^{mito}(x_{FCS}) = S_T^{FRAP}$. We do not know what the binding sites are but let us assume that they are uniformly distributed along the embryo, *i.e.*, $S_T^{intercyt}(x) = S_T^{c,FCS}$ and $S_T^{mito}(x) = S_T^{FRAP}$ $\forall x$ along the axis. Under this assumption we know $S_T^{intercyt}/Bcd_T^{c,FCS}$ and $S_T^{mito}/Bcd_T^{c,FCS}$ everywhere along the embryo. We also know $K_D/Bcd_T^{c,FCS}$ which we assume is constant and uniform. Let us define $\zeta(x) \equiv Bcd_T(x)/Bcd_T^{c,FCS}$ which can correspond to either interphase or mitosis depending on whether the value of $Bcd_T(x)$ corresponds to interphase or mitosis. Then, inserting $S_T^{intercyt}/Bcd_T^{c,FCS}$, $K_D/Bcd_T^{c,FCS}$ and ζ in Eqs. (2)–(4) we can compute the ratios with respect to $Bcd_T^{c,FCS}$ of all relevant cytoplasmic concentrations during interphase as functions of ζ. Analogously, we can use $S_T^{mito}/Bcd_T^{c,FCS}$, $K_D/Bcd_T^{c,FCS}$ and ζ to compute similar ratios but for the concentrations during mitosis. Given these ratios, we can also compute the corresponding D_{sm} and D_{coll} as functions of ζ using Eqs. (8)–(9). We show in the main body of

the paper plots of $D_{sm}(S_T^{mito},\zeta)$, $D_{sm}(S_T^{intercyt},\zeta)$ and $D_{coll}(S_T^{intercyt},\zeta)$ obtained in this way.

X. ANALYSIS OF SOME APPROXIMATIONS OF OUR MODEL

A. Timescales.

The analytic expressions of the ACF that we use in this paper are derived under the assumption that the transport of Bcd across the illumination volume can be described in terms of effective diffusion coefficients. This assumption holds when reactions occur over a faster timescale than diffusion in the observation volume [4, 14]. The shortest diffusion timescale of the problem is the one associated to the free diffusion of Bcd. Considering a mean, ℓ, between the two lengthscales of the observation volume, w_r and w_z, *i.e.* $\ell \approx 1.2\mu m$, we obtain $\tau_D = \ell^2/(4D_f) = 0.02s$ for the shortest diffusion timescale. The reaction timescale, τ_r, can be defined as $\tau_r^{-1} = k_{off}(1 + Bcd_{feq}/K_D + S_{eq}/K_D)$. Using the values of Table VI we obtain: $\tau_r = 1/(92k_{off})$ and $\tau_r = 1/(146k_{off})$ for Case II in the cytoplasm and the nucleus, respectively and $\tau_r = 1/(44k_{off})$ for Case III in the cytoplasm. If $\tau_r < 0.1\tau_D$ the fast reaction approximation holds very well. Even $\tau_r \sim \tau_D$, the residence times associated to effective coefficients can be derived from the ACF [14]. Thus, if $k_{off} > 10/s$ we can be certain that the fast reaction approximation holds even in the worst case scenario ($\tau_r = 1/(44k_{off})$), but the estimates should still be good even if $k_{off} \sim 1.2/s$.

B. FRAP and the corona effect.

The recovery time derived from FRAP in [1] is of the same order of magnitude as the time it takes to photobleach the observation volume. This means that once the photobleaching is over and the recovery is monitored there is a noticeable fraction of bleached molecules outside the observation volume. This leads to the so-called *corona* effect [13]. If the data is fitted as if the fraction of bleached molecules outside the observation volume were negligible the recovery time and, consequently, the diffusion coefficient, are understimated [15]. In [15] there is a discussion on how to analyze the data and yet extract meaningful transport rate estimates. In any case, it could still be good to analyze the data under the approximation of an instantaneous bleaching pulse as done in [1]. In order to test to what extent this effect would affect the diffusion coefficient estimate in a situation like the one described by our model we performed numerical simulations of equations of the form (26)–(29) but with additional terms to take into account the conversion of the tagged species into untagged ones. Namely, we added the terms: $-\omega_B Bcd_f^t$, $-\omega_B Bcd_b^t$, $\omega_B Bcd_f^t$ and $\omega_B Bcd_b^t$, to Eqs. (26)–(29), respectively, with $\omega_B \neq 0$ only for $r \leq r_B = 0.95\mu m$ and during the duration of the bleaching pulse ($t \leq t_B$). For the bleaching pulse we used the values $t_B = 0.21$, 0.45 and $1s$ and chose ω_B so that the fluorescence depletion at $t = 1s$ for the case with $t_B = 1s$ was similar to the value displayed in Fig. S5 of [1] for a similar bleaching pulse duration. For the initial condition we used:

$$Bcd_f^t = Bcd_{feq},\ Bcd_b^t = Bcd_{beq},\ Bcd_f^u = Bcd_b^u = 0, \tag{43}$$

with the values of Case III listed in Table VII and, correspondingly, the dissociation constant and free diffusion coefficients of Case III, $K_D = 0.0026$, $D_f = 19\mu m^2/s$, $D_S = 0.095\mu m^2/s$. Thus, we used concentrations and the dissociation constant in units of $Bcd_T^{c,FCS}$. We also used $k_{off} = 10/s$ to guarantee the validity of the fast reaction limit (reaction dominant case of [3]). We computed the normalized fluorescence, $F_n(t)$, inside the sphere of radius r_B as a function of time:

$$F_n(t) = \frac{\int_0^{r_B} dr\, r^2 \left(Bcd_f^t(\mathbf{r},t) + Bcd_b^t(\mathbf{r},t)\right)}{\int_0^{r_B} dr\, r^2 \left(Bcd_f^t(\mathbf{r},t=0) + Bcd_b^t(\mathbf{r},t=0)\right)}, \tag{44}$$

for the simulations with $t_B = 0.21$, 0.45 and $1s$ and for one representing a situation of instantaneous bleaching ($t_B = 0$). For the latter, we numerically integrated Eqs. (26)–(29) with the initial condition:

$$\begin{aligned} Bcd_f^t = Bcd_{feq},\ Bcd_b^t = Bcd_{beq}, \quad & Bcd_f^u = Bcd_b^u = 0, \\ & \text{for } r \geq r_B; \\ Bcd_f^t = \delta_B Bcd_{feq},\ Bcd_b^t \ =\ & \delta_B Bcd_{beq}, \\ Bcd_f^u = (1-\delta_B) Bcd_{feq},\ Bcd_b^u \ =\ & (1-\delta_B) Bcd_{beq}, \\ & \text{for } r < r_B; \end{aligned} \tag{45}$$

with the equilibrium values as in the simulations with finite bleaching duration and with δ_B such that the integral in the numerator of Eq. (44) at $t = 0$ had the same value as the one obtained with $t_B = 1s$ at $t = 1s$ (*i.e.*, at the end of the 1s bleaching pulse). We show with dashed lines in Fig. S1 the time courses obtained in all four cases. In this figure we also show with solid lines fits to the curves of the form

$$F_{fit}(t) = A - B\exp(-t/t_c). \tag{46}$$

From these fits we obtain the following half recovery times ($t_{1/2} \equiv t_c \ln(2)$) in seconds: 0.18 ± 0.002, 0.22 ± 0.002, 0.26 ± 0.002 and 0.29 ± 0.003 for $t_B = 0$, 0.21, 0.45 and $1s$, respectively. Thus, in these simulations the half recovery time increases by a factor of 1.3 when changing the bleaching duration from 0.21s to 1s, while it increases by a factor ~ 1.2 when going from the instantaneous bleaching to $t_B = 0.21s$. In the experiments of [1] it is $t_{1/2} \sim 0.21s$ and $t_{1/2} \sim 0.55s$ for the smallest ($t_b \sim 0.35s$) and the largest ($t_b \sim 1.05s$) values of t_b probed, respectively. Thus, it increases by a factor of ~ 2.5 when t_b increases by a factor of 3. Based on our

simulations, we do not expect that the half recovery time can decrease by an order of magnitude with respect to the case with $t_b \sim 0.35s$ if an experiment with instantaneous bleaching could be performed. Applying the relationship $D = 0.224w^2/t_{1/2}$ to our simulation results, on the other hand, gives $D = 1.16$, 0.92, 0.81 and $0.7\mu m^2/s$, for $t_B = 0$, 0.21, 0.45 and $1s$, respectively, which is of the order of magnitude of D_{sm} at the conditions of the simulation ($D_{sm} \approx 1\mu m^2/s$).

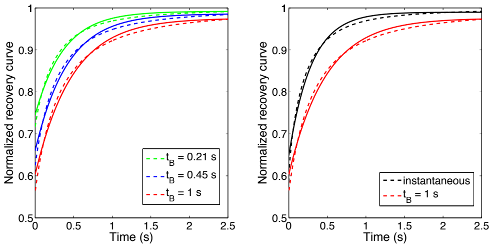

FIG. 1: **Recovery curves from simulations of FRAP experiments.** The dashed curves correspond to the results of the simulations and the solid ones to fits of the form (46). (A) Recovery curves for finite bleaching durations, $t_B = 0.21$, 0.45 and $1s$. (B) Comparison of the recovery curves for $t_B = 1s$ and for a situation with instantaneous bleaching.

C. Cooperative vs non-cooperative binding to sites.

Our model is very simplified regarding binding since it assumes that the binding sites act independently of one another. It has been determined that Bcd binds cooperatively to multiple sites of DNA [16]. In order to modify our model so as to include cooperativity we would have to replace the scheme (1) by one with multiple reaction steps. The model would be very complicated and it would be impossible with the experimental data that we have used here to quantify the parameters of a biophysical model. Our simple model should then be considered as providing some sort of "effective" description. One could then ask in which ways the estimates derived from the experiments would change if we used a binding model that included cooperativity. The simplest way to approach this question is to consider a scheme with very high cooperativity between two binding sites, so that the reaction between Bcd and its binding sites can be modeled by:

$$2Bcd_f + S \underset{\hat{k}_{off}}{\overset{\hat{k}_{on}}{\rightleftarrows}} Bcd_b. \qquad (47)$$

In such a case, the collective effective diffusion coefficient is given by:

$$D_{coll} = \frac{D_f + \frac{2\hat{B}cd_{feq}\hat{S}_{eq}^2}{S_T\hat{K}_D^2} D_S}{1 + 2\hat{B}cd_{feq}\hat{S}_{eq}^2/S_T\hat{K}_D^2}, \qquad (48)$$

with $\hat{K}_D^2 = \hat{k}_{off}/\hat{k}_{on}$ and $\hat{B}cd_{feq}$ and $\hat{S}_{eq}$ the equilibrium concentration values of free Bcd and free binding sites in the case of scheme (47). Given Eq. (48) we conclude that the quantity $\gamma = (D_f - D_{coll})/(D_{coll} - D_S)$ which is given by Eq. (37) in the case of indenpendent binding sites would be given by:

$$\gamma = \frac{2\hat{B}cd_{feq}\hat{S}_{eq}^2}{S_T\hat{K}_D^2}, \qquad (49)$$

in the case of the scheme (47). If we use the experimental parameter values of Case III, for example, we obtain $\gamma = (D_f - D_{coll})/(D_{coll} - D_S) = 0.36$, which corresponds to $S_{eq}^2/(S_T K_D)$ in the case of independent binding sites and to $2\hat{B}cd_{feq}\hat{S}_{eq}^2/(S_T\hat{K}_D^2)$ in the case of scheme (47). This implies that the values that we would derive if we used the cooperative scheme would be approximately related to those obtained in the case of independent binding by: $\hat{B}cd_{feq}\hat{S}_{eq}/\hat{K}_D^2 \sim S_{eq}/K_D$. Given that Bcd_{feq}/K_D and S_{eq}/K_D are approximately of the same order of magnitude in Case III (See Table VI), we can get a very rough estimate by doing $\hat{B}cd_{feq}/\hat{K}_D \sim \sqrt{Bcd_{feq}/K_D}$ and $\hat{S}_{eq}/\hat{K}_D \sim \sqrt{S_{eq}/K_D}$. Based on this analysis we expect the estimate of the dissociation constant that would be derived for the cooperative scheme (47) to be larger than the one derived in the independent case ($\hat{K}_D/Bcd_T \sim \sqrt{K_D/Bcd_T} \sim 0.05$). Inserting in $\hat{K}_D/Bcd_T \sim 0.05$ the dissociation constant estimated in the experiments that proved that Bcd binds cooperatively to multiple sites of DNA [16] ($\hat{K}_D \sim 5nM$) we obtain $Bcd_T \sim 100nM$ which is a reasonable value. In order to obtain more accurate results, however, we should study the net transport that results in the case of scheme (47) in more detail. This study is not completely trivial and will be left for a future work.

XI. FROM RATIOS TO ABSOLUTE CONCENTRATIONS.

In order to infer absolute concentrations from the ratios of concentrations estimated in the previous Sections we need to know $[Bcd_T]$. Optical experiments can provide estimates of the concentration of fluorescent Bcd, $[Bcd_T^t]$. Thus, it is necessary to know the fraction of fluorescent to total Bcd concentration to go from the ratios of Table VI to absolute values. In principle, this fraction could be derived from some of the weights of the terms of the ACF (see Eqs. (17)). However, we have avoided using the weights because we think there are some uncertainties on how they should be modelled. There is, on one hand, the potential problem associated to photobleaching. Then, there is the problem of what is the correct statistics that provides a reliable analytical expression of the weights. The fraction of mature EGFP has been estimated in [8] comparing the fluorescence intensity in fixed embryos (where it is expected that all EGFP be mature)

with that observed in live embryos (where only a fraction is mature and thus, fluorescent). The observation of fixed samples introduces an intensity increase *per se* which the authors estimate as a factor of 3 using fluorescent proteins that mature very fast (Fig. 8F of [8]). Taking this factor into account, the fraction of fluorescent to total Bcd-EGFP concentration, f, was estimated to be $\sim 55--60\%$ at the tip of the anterior pole (where f is smallest) and achieve $\sim 100\%$ at a distance of the order of 20% of the embryo's length or larger (Fig. 8G of [8]). There is an uncertainty then on what is the fraction that should be considered at the region where the FCS experiments were performed. Combining Eqs. (17) and (24) we obtain $G_{sm}/Go_T = (1-f)$. Thus, f can be derived from the relative weight of the second component of the ACF ($F^{(2)}$ in the results derived using Bcd-EGFP as listed in Tables I and III for the cytoplasm and nuclei, respectively). Eqs. (17) and (24) correspond to Case III and we obtain $f = 0.68$. If we assume that the same relation also holds for Case II, we obtain $f = 0.82$ in the cytoplasm and $f = 0.58$ in nuclei. If we consider Eqs. (20)–(22) which are the ones that correspond to $D_S = 0$ (Case II) then we can equate the ratio Go_{sm}/Go_{coll} to that of the relative weights $F^{(2)}/F^{(1)}$ obtained experimentally (Table I). We find $f = 0.99$ and $f = 0.98$ using the results obtained in the cytoplasm and in nuclei, respectively. These are very rough estimates given that they were obtained using expressions that hold if the traps, S, are exactly immobile. In particular, this is not the situation assumed for Case III. Knowing f and the concentration of fluorescent Bcd (Table IV) we can go from ratios of concentrations to absolute values as displayed in Tables VIII and IX. In order to obtain the estimates of these tables we have used $f = 0.8$ which is some sort of mean of the very rough estimates obtained for Cases II and III assuming a Poisson statistics for all variables and a multinomial statistics for Bcd_b^t, Bcd_b^u and S. For the concentrations, we have probed the two extreme values of fluorescent Bcd estimated in nuclei in [1] (19nM and 140nM) and then derived Bcd_T^t assuming that K_D is the same in nuclei and in the cytoplasm for Case II. In any case, the uncertainties on the weight expressions and on the total number of fluorescent molecules estimated in [1] points to the need of considering the Tables VIII and IX with caution.

[1] Abu-Arish A, Porcher A, Czerwonka A, Dostatni N, Fradin C (2010) High mobility of bicoid captured by fluorescence correlation spectroscopy: Implication for the rapid establishment of its gradient. Biophysical Journal 99: L33 - L35.

[2] Pando B, Dawson SP, Mak DOD, Pearson JE (2006) Messages diffuse faster than messengers. Proc Natl Acad Sci (USA) 103: 5338-5342.

[3] Sprague BL, Pego RL, Stavreva DA, McNally JG (2004) Analysis of binding reactions by fluorescence recovery after photobleaching. Biophysical Journal 86: 3473 - 3495.

[4] Sigaut L, Ponce ML, Colman-Lerner A, Dawson SP (2010) Optical techniques provide information on various effective diffusion coefficients in the presence of traps. Phys Rev E 82: 051912.

[5] Krichevsky O, Bonnet G (2002) Fluorescence correlation spectroscopy: the technique and its applications. Reports on Progress in Physics 65: 251-297.

[6] Thompson NL, Navaratnarajah P, Wang X (2011) Measuring surface binding thermodynamics and kinetics by using total internal reflection with fluorescence correlation spectroscopy: Practical considerations. The Journal of Physical Chemistry B 115: 120-131.

[7] Pérez Ipiña E, Ponce Dawson S (2014) Fluctuations, correlations and transport inside cells. To be published.

[8] Little SC, Tkaik G, Kneeland TB, Wieschaus EF, Gregor T (2011) The formation of the bicoid morphogen gradient requires protein movement from anteriorly localized mrna. PLoS Biol 9: e1000596.

[9] Porcher A, Abu-Arish A, Huart S, Roelens B, Fradin C, et al. (2010) The time to measure positional information: maternal hunchback is required for the synchrony of the Bicoid transcriptional response at the onset of zygotic transcription. Development 137: 2795-804.

TABLE VIII: Parameter estimates that correspond to the cytoplasm and the nucleus during interphase obtained using information on the concentration of Bcd-EGFP molecules and assuming $f = 0.8$ and that K_D has the same value in both regions. Case II.

**Case II, Interphase, FCS location**				
	**Cytoplasm**		**Nucleus**	
[Bcd-egfp](nM)	7.97	58.75	19	140
S_{eq} (nM)	0.23	1.71	0.43	3.13
Bcd_{beq} (nM)	9.77	71.96	23.49	173.1
Bcd_{feq} (nM)	0.20	1.47	0.26	1.93
K_D (nM)	$5\,10^{-3}$	0.035	$5\,10^{-3}$	0.035
S_T (nM)	10	73.67	24	176.2
Bcd_T (nM)	9.97	73.4	23.75	175

TABLE IX: Parameter estimates that correspond to the cytoplasm during interphase obtained using the values of [Bcd-egfp] derived for Case II and assuming $f = 0.8$. Case III.

**Case III, Interphase, FCS location**		
	**Cytoplasm**	
[Bcd-egfp](nM)	7.97	58.75
S_{eq} (nM)	0.29	2.17
Bcd_{beq} (nM)	9.17	67.59
Bcd_{feq} (nM)	0.79	5.85
K_D (nM)	0.03	0.19
S_T (nM)	9.47	69.76
Bcd_T (nM)	9.97	73.43

[10] Gregor T, Wieschaus EF, McGregor AP, Bialek W, Tank DW (2007) Stability and nuclear dynamics of the bicoid

morphogen gradient. Cell 130: 141 - 152.

[11] Brown EB, Wu ES, Zipfel W, Webb WW (1999) Measurement of molecular diffusion in solution by multiphoton fluorescence photobleaching recovery. Biophysical Journal 77: 2837 - 2849.

[12] Soumpasis D (1983) Theoretical analysis of fluorescence photobleaching recovery experiments. Biophys J 41: 95–97.

[13] Weiss M (2004) Challenges and artifacts in quantitative photobleaching experiments. Traffic 5: 662–671.

[14] Ipiña EP, Dawson SP (2013) From free to effective diffusion coefficients in fluorescence correlation spectroscopy experiments. Phys Rev E 87: 022706.

[15] Braga J, Desterro JM, Carmo-Fonseca M (2004) Intracellular macromolecular mobility measured by fluorescence recovery after photobleaching with confocal laser scanning microscopes. Molecular Biology of the Cell 15: 4749-4760.

[16] Ma X, Yuan D, Diepold K, Scarborough T, Ma J (1996) The drosophila morphogenetic protein bicoid binds dna cooperatively. Development 122: 1195-1206.
