# Supplementary material for: Messages Do Diffuse Faster than Messengers: Reconciling Disparate Estimates of the Morphogen Bicoid Diffusion Coefficient
Source: PLoS Comput Biol. 2014 Jun 5;10(6):e1003629. doi: 10.1371/journal.pcbi.1003629 (PMC4046929; doi:10.1371/journal.pcbi.1003629)
Supplement: Text S3 — Mean square displacement and second moments of particle distributions. Here we describe the meaning of the various diagnostics that we perform on the particle simulations. In particular, we show how the slope of the MSD and of the different second moments, , that we compute are related to the different diffusion coefficients that we discuss in this paper. (PDF) [file pcbi.1003629.s003.pdf]

Text S3. Mean square displacement and second moments of particle distributions.

We compute the mean square displacement (**MSD**) as:

$$\langle r^2\rangle_\Sigma = \frac{1}{N}\sum_{i=1}^{N}(x_i(t)-x_{i0})^2 \qquad (1)$$

where the subscript, i, refers to each particle for which the mean square displacement is computed, $x_i(t)$ and x_{i0} are the i-th particle positions (in three space dimensions) at time, t, and at the initial time, respectively, and N is the total number of particles over which the sum is performed. In all the particle simulations performed in this paper $\langle r^2\rangle_\Sigma$ eventually scales linearly with time

$$\langle r^2\rangle_\Sigma = 2dDt \qquad (2)$$

with D a diffusion coefficient and $d = 3$ the number of space dimensions in the simulations.

For the simulations with added particles we also compute the second moment of the particles distribution. This is obtained by performing an integral: $\int_V r^2\rho_X(r,t)dV$ over the simulation volume where ρ_X is proportional to the concentration of molecules of type X at position r and time t. In this expression r is the d-dimensional position measured from the origin ($d = 3$). In all the cases that we consider in the paper, the deviation of ρ_X with respect to a spatially uniform equilibrium value, *i.e.* $\delta\rho_X \equiv \rho_X - \rho_{Xeq}$, eventually is governed by the diffusion equation:

$$\frac{\partial\delta\rho_X}{\partial t} = D\nabla^2\delta\rho_X\ . \qquad (3)$$

Then, if $\delta\rho_X$ is negligible at the borders of a volume V, the integral $\int_V r^2\delta\rho_X dV$ scales linearly with time with a constant of proportionality that depends on D as we show now. To this end, we multiply both sides of Eq. (3) by r^2 and integrate over space to obtain:

$$\frac{d}{dt}\int_V r^2\delta\rho_X dV = 2Dd\int_V \delta\rho_X dV. \qquad (4)$$

For this derivation, the divergence theorem has been used twice and boundary terms neglected. The latter implies that, at the border of the volume, $\hat{n}\cdot\nabla\delta\rho_X = 0$ with $\hat{n}$ the normal to the border and that the integral of $r\delta\rho_X$ over the volume's surface be negligible with respect to $\int_V \delta\rho_X dV$. The former is satisfied for no-flux boundary conditions and the latter pertains when the perturbation is due to the spread of a bolus of particles on top of a spatially uniform background as considered in the particle simulations. Given that $\hat{n}\cdot\nabla\delta\rho_X = 0$ at the border, $\int_V \delta\rho_X dV$ is a constant. We then conclude that

$$\frac{\int_V r^2\delta\rho_X dV}{\int_V \delta\rho_X dV} = 2Ddt + \overline{K} \qquad (5)$$

where $\overline{K}$ is a constant. Now, given that $\int_V r^2\delta\rho_X dV = \int_V r^2(\rho_X - \rho_{Xeq})dV$ and that $\int_V r^2\rho_{Xeq}dV$ is a constant, we then conclude that:

$$\langle r^2\rangle_{\int X} \equiv \frac{\int_V r^2\rho_X dV}{\int_V \delta\rho_X dV} = 2Ddt + K \qquad (6)$$

with another constant, K. This is the second moment that we compute using all the particles or only the added (or fluorescent) ones in the added bolus simulations. We approximate the integrals by partitioning space into boxes and counting the particles in each box. We denote the number of particles in the ith box by n_i. The squared distance of the geometric center of the ith box from the origin is denoted r_i^2. Then we approximate $\langle r^2\rangle_{\int X}$ by:

$$\langle r^2\rangle_{\int X} \approx \frac{1}{N}\sum_i n_i r_i^2 \qquad (7)$$

where N is the number of added particles. When X refers to the fluorescent (*i.e.* added) particles, n_i is the number of fluorescent particles in the ith box at each time and it is the number of all particles in this box when X refers to both fluorescent and non-fluorescent (*i.e.* all) particles.

In the paper we present the results of three sets of particle simulations: (1)Free particles in the absence of binding sites, (2)Simulated **FRAP**-like experiment and (3)Simulated particle bolus experiment. In the last two sets binding sites are present and free particles react with them. We compute the **MSD** for the three sets. In the absence of binding sites Eq. (2) holds with $D = D_f$, the free diffusion coefficient of the particles. When binding sites are present, the diffusion coefficient that enters Eq. (2) is $D = D_{sm}$ as shown in [1]. We compute the second moment, $\langle r^2\rangle_{\int X}$ for the added bolus simulations both in the presence and in the absence of binding sites. We do this both for all the particles ($X = all$) and just for the added ones ($X = fluo$). In the absence of binding sites the diffusion coefficient that enters Eq. (6) is $D = D_f$ both for $X = all$ and $X = fluo$. As shown in [1], in the presence of binding sites, the perturbation with respect to equilibrium eventually evolves according to Eq. (3) with $D = D_{coll}$ for $X = all$ and with $D = D_{sm}$ for $X = fluo$.

---

[1] B. Pando, S. Ponce Dawson, D. D. Mak and J. Pearson, Proc. Natl. Acad. Sci. U.S.A. **103** (14), 5338 – 5342 (2006).
